# Low Carbohydrate Dietary Approaches for People With Type 2 Diabetes—A Narrative Review

**DOI:** 10.3389/fnut.2021.687658

**Published:** 2021-07-15

**Authors:** Sean D. Wheatley, Trudi A. Deakin, Nicola C. Arjomandkhah, Paul B. Hollinrake, Trudi E. Reeves

**Affiliations:** ^1^X-PERT Health, Hebden Bridge, United Kingdom; ^2^School of Social and Health Sciences, Leeds Trinity University, Leeds, United Kingdom

**Keywords:** low carbohydrate diets, carbohydrate restriction, Type 2 diabetes, nutrition, blood glucose control

## Abstract

Although carbohydrate restriction is not a new approach for the management of Type 2 diabetes, interest in its safety and efficacy has increased significantly in recent years. The purpose of the current narrative review is to summarise the key relevant research and practical considerations in this area, as well as to explore some of the common concerns expressed in relation to the use of such approaches. There is a strong physiological rationale supporting the role of carbohydrate restriction for the management of Type 2 diabetes, and available evidence suggests that low carbohydrate dietary approaches (LCDs) are as effective as, or superior to, other dietary approaches for its management. Importantly, LCDs appear to be more effective than other dietary approaches for facilitating a reduction in the requirement for certain medications, which leads to their effects on other health markers being underestimated. LCDs have also been demonstrated to be an effective method for achieving remission of Type 2 diabetes for some people. The available evidence does not support concerns that LCDs increase the risk of cardiovascular disease, that such approaches increase the risk of nutrient deficiencies, or that they are more difficult to adhere to than other dietary approaches. A growing number of organisations support the use of LCDs as a suitable choice for individuals with Type 2 diabetes.

## Introduction

The use of low carbohydrate dietary approaches (LCDs) in people with Type 2 diabetes is not new, but standard care around much of the world has focused on the use of a low fat, calorie controlled diet—in line with that usually recommended for the general population—for much of the last few decades. Recently however, LCDs have gained in popularity again. As a result, interest in their safety, efficacy and effectiveness has increased. Despite such diets being used by an increasing number of people with Type 2 diabetes, the use of carbohydrate restriction in this population is still not without controversy. In fact, commentary on this approach has often been hostile, with much of the criticism being inappropriate and failing to objectively consider the available evidence. The purpose of the current narrative review is therefore to consider the existing body of research, and to explore common concerns and practical considerations associated with the use of this approach. The review and its recommendations are aimed at both clinicians, to help guide decision making, and researchers within this area, to summarise the existing evidence and help guide future research directions.

## What Are Low Carbohydrate Dietary Approaches?

**Figure 1:**
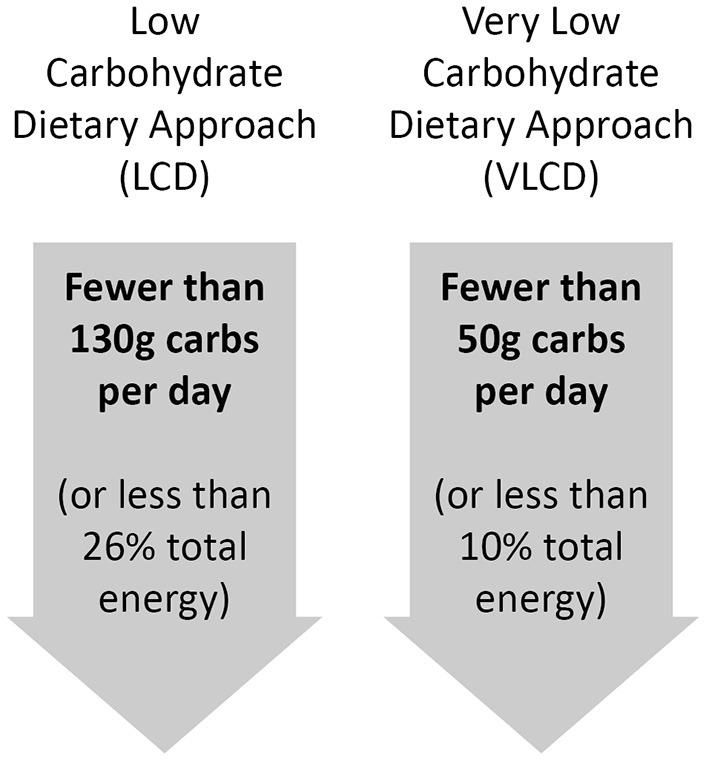
Suggested definitions for low and very low carbohydrate dietary approaches.

Although there is no universally agreed definition of what constitutes a LCD, the most commonly used definitions are those outlined in Figure 1, consistent with those proposed by Feinman et al. (1).

It can be argued that the definition of any diet is somewhat subjective, and often ambiguous. It is therefore important that, where there is any uncertainty, the definitions used to consider the impact of any given diet is based on those applied by people who work with that approach in the field. This is true of the definitions of LCD used here, which come from specialists in the field and are well-represented within the references used in their review (1). These definitions will therefore be applied throughout the current review.

It is important that the appraisal of available evidence considers these definitions appropriately. It is common for research, both in the form of trials and observational studies, to use terminology such as “low carbohydrate diet scores” or to refer to “low carbohydrate diet patterns” when the carbohydrate intake of individuals within the study far exceeds the thresholds stated here. This is inappropriate and misleading, and such studies should not be used to draw conclusions regarding the safety and efficacy of LCDs.

## Physiological Rationale for, and Possible Benefits of, Carbohydrate Restriction

**Figure 2:**
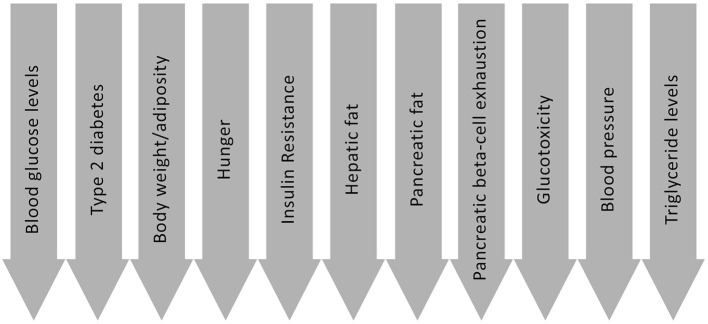
Possible benefits of carbohydrate restriction for people with Type 2 diabetes.

There are a number of routes through which LCDs may be beneficial for people with Type 2 diabetes; the most significant of which are introduced below, and are summarised in Figure 2. These are not all necessarily exclusive to LCDs, but nevertheless are means by which dietary approaches of this nature may be effective. Possible mechanisms and effects include:

- **Improved blood glucose control (and possible remission of Type 2 diabetes)—**Most people with Type 2 diabetes have a reduced ability to remove carbohydrate from their blood efficiently, as a result of insulin resistance (2, 3). They also often have an impaired ability to moderate the delivery of new glucose into the blood, as the body is less able to control gluconeogenesis (4). The absence of the first phase insulin response, a pathology that is typical of Type 2 diabetes (5, 6), further exacerbates this latter issue, because this impairs the body's ability to prevent glucose being released from the liver when glucose is entering the circulation from dietary sources (7). Reducing the intake of dietary carbohydrate, the nutrient that has the biggest impact on glycaemic control (8), can mitigate for these issues, leading to rapid improvements in blood glucose control even before any reduction in body weight is seen (9). That carbohydrate restriction is the most effective method for lowering blood glucose levels should not be considered controversial, and is supported by a recent American Diabetes Association (ADA) position statement which asserted that “Reducing overall carbohydrate intake for individuals with diabetes has demonstrated the most evidence for improving glycaemia” (10). The acute effect of LCDs on blood glucose levels is further demonstrated by the fact glucose lowering agents need to be reduced at the onset of a LCD (11). In addition to the acute effects on blood glucose control, LCDs may also help to address some of the underlying causative factors, as outlined below. Linked to these benefits, there is evidence that some people may be able to put their Type 2 diabetes into remission through following a LCD (12). This is discussed in more detail later in the review.
- **Improved weight management—**Although some of the benefits of LCDs may be independent of weight loss (13, 14), many of the possible positive effects are likely influenced or caused by a reduction in body fat (15). There are a number of mechanisms through which LCDs may improve weight management, the most important of which is perhaps the commonly seen reduction in *ad libitum* energy intake in individuals following the adoption of a LCD (16–19). Supporting the presence of this effect, some studies comparing dietary approaches allow *ad libitum* energy intake in LCD groups whilst imposing an explicit calorie restriction on the control groups [e.g., (20–24)]. Reduced hunger, the likely mechanism explaining these observations, is discussed further below.
- It should however be noted that a decrease in *ad libitum* energy intake compared to other dietary approaches is not a universal observation. For example, a recent highly controlled metabolic ward study comparing a low carbohydrate animal based diet with a low fat plant based diet observed a lower energy intake in the latter group (25). The short-term nature of this study may have played a role in these findings however, with evidence suggesting an adaptation period may be required before the true impact of carb restriction can be observed (26). It is also important to note that both of the diets were based on high quality, minimally processed foods; i.e., the diet LCD was being compared to was not necessarily indicative of a standard Western diet. Any differences between the two diets do not therefore necessarily suggest a lack of efficacy or effectiveness on either of their behalves, but rather both diets were likely health promoting in comparison to the standard eating pattern of many people.
- A second potential contributor is that LCDs naturally result in a reduced intake of the types of foods that are often considered to be highly processed, or “ultra-processed.” Foods that are categorised in this way usually combine fats with carbohydrates, and contain high concentrations of salt, sugar and/or chemical preservatives or flavour enhancers. The presence of some of these ingredients, and/or the combination in which they are present in such foods, is posited to have an undesirable effect on health. The avoidance of ultra-processed foods is an inevitable outcome of following a LCD, as these products invariably contain large quantities of carbohydrates and thus would be incompatible with this way of eating. This is significant in relation to weight management, as consumption of these energy-dense, nutrient poor foods has been shown to result in increased energy intake and, consequently, weight gain (27).
- A further point that is pertinent to the issue of weight management is that LCDs are effective for reducing insulin levels and insulin resistance (16, 28–31). Recent evidence suggests that an increase in insulin levels may precede obesity, rather than the converse (32). There are a number of pathways through which this may be affected. Insulin's most sensitive effect is the inhibition of lipolysis, whilst it also increases lipogenesis significantly (33). Insulin therefore shifts energy partitioning within the body toward fat storage. Insulin interacts with other regulatory factors too—including other hormones, neuronal activity and gut function—and so may influence weight management through multiple means (33). Reducing insulin levels in Type 2 diabetes, which is a hyperinsulinaemic condition (34, 35), should therefore be considered a priority (9). Further, weight gain is consistently seen in those on insulin therapy (36). The impact of insulin on weight management in this population may be even greater than in those who do not take exogenous insulin, as 50–80% of insulin produced in the pancreas is taken up by the liver cells whereas a greater proportion of injected insulin is circulated around the body, increasing the storage of fat (33). Therefore, the ability of LCDs to reduce insulin requirements in those who use exogenous insulin (11, 37, 38) can have important benefits on this front.
- LCDs may also increase energy expenditure, which would contribute to improved weight management. A recent meta-analysis considered this question, and concluded that although in short-term studies (<2.5 weeks) total energy expenditure was reduced in LCDs, in longer studies there was a small but significant increase in total energy expenditure (26). This finding also highlights the importance of appraising short-term studies of LCDs carefully, as their impact may change temporally, potentially reflecting the need for individuals to be given chance to adapt to a LCD when they first begin one.
- **Reduced hunger—**As alluded to previously, reduced hunger is often reported in individuals who follow a LCD (39). Possible reasons for this include an increase in energy availability in the late post-prandial period (40, 41) and/or changes in hunger hormones, such as a reduction in ghrelin (42). It may also in part be due to the improvement in the quality of the foods people tend to eat when adopting this way of eating. This is not a trait that is necessarily unique to carbohydrate restriction, but, as noted above, omitting carbohydrate-rich foods naturally involves cutting out many of the highly processed products some people regularly consume (for example cakes and crisps). These foods are often energy dense and hyper-palatable, causing people to crave more of them (43, 44); an issue that is avoided when higher quality foods are consumed in preference. Adopting a LCD may also lead to an increased intake of protein, which has consistently been shown to be the macronutrient that has the greatest influence on satiety (45, 46) and has even been posited to be central in regards to the link between diet and obesity (47, 48).
- In those following a VLCD, hunger may be further reduced due to the onset of nutritional ketosis (16–18, 49, 50). Supporting the significance of this effect, the influence of ketosis on appetite suppression is believed to play an important role in the efficacy of very low energy diets too (51). Nutritional ketosis should not be confused with diabetic ketoacidosis (DKA), which is a life-threatening condition caused by insulin insufficiency. DKA is mostly seen in people with Type 1 diabetes, though occasionally occurs in people with Type 2 diabetes, and requires urgent hospital treatment to address the dehydration and lowered blood pH caused by a large increase in ketone levels. However, in comparison, nutritional ketosis is a natural process that occurs when someone is utilising fat as their primary fuel source. This has not been shown to be dangerous as long as some insulin is available and blood glucose levels are not elevated (52).
- **Reduced insulin resistance—**Most people with Type 2 diabetes have a high degree of insulin resistance (2, 3), and there is evidence demonstrating that changes in insulin resistance can predict the onset of diabetes (53). Insulin resistance is a complex subject however, and a full exploration of the nuance of this topic is beyond the scope of the current review. This section will therefore focus on the potential therapeutic benefits of LCDs for addressing pathological insulin resistance broadly, without considering the intricacies of this. It is however noteworthy that physiological (i.e., non-pathological) insulin resistance may also occur in response to a LCD as an adaptive response to prevent hypoglycaemia, and may even be site specific to allow glucose dependent cells (such as those in the brain or kidneys for example) priority usage of available glucose. More research is required in this area.
- The primary means through which pathological insulin resistance can be addressed appears to be through fat loss, particularly from the central organs (54), which can be facilitated by a LCD. Fat loss from the liver, discussed subsequently, reduces its resistance to insulin (54); leading to a reduction in gluconeogenesis (7) and important benefits for overall blood glucose control. A second pathway through which a LCD may help to address pathological insulin resistance is through reducing the body's exposure to insulin (55–57); thus this dietary approach can help to address this key issue through multiple means.
- Although Type 2 diabetes is often considered to be a condition of insulin resistance, an alternative interpretation of the available data is that it is the high insulin levels themselves (i.e., hyperinsulinaemia) that are the primary issue (58). Regardless of the underlying pathology, the ability of a LCD to reduce insulin levels, and thus exposure to insulin, is likely beneficial.
- **Reduced hepatic fat storage—**Elevated fat storage in the liver is a key driver of Type 2 diabetes (59), with hyperinsulinaemia and excess energy intake thought to be causative in the development of hepatic fat accumulation (60, 61). Although weight loss can be effective for reducing liver fat content, there is evidence that the benefits of LCDs on this front may be, at least in part, independent of this (62–65); and that greater improvements may be achieved through carbohydrate restriction than through calorie restriction alone (61, 66). Research has also shown that improvements in response to LCDs can occur rapidly (67). Overconsumption of carbohydrate, when compared to other nutrients, may be especially detrimental to the liver (60, 61), with excess being converted to fat through *de novo lipogenesis* (68). Further still, sugary carbohydrates may be particularly harmful because the majority of fructose that enters the body has to be processed within the liver before it can be stored or utilised by other cells (69). Based on this, the ability of LCDs to reduce liver fat is perhaps not surprising as they can address both of the primary mechanisms of hepatic fat accumulation; i.e., elevated insulin levels and excess energy intake (particularly from carbohydrates).
- **Reduced pancreatic fat storage—**A reduction in hepatic fat also helps to facilitate a reduction of fat in the pancreas. Triglyceride rich lipoproteins expelled from the liver have a direct impact on the pancreas, as outlined in Professor Roy Taylor's twin-cycle hypothesis (59), supported by recent findings from the Diabetes Remission Clinical Trial (DiRECT) (70). A reduction in hepatic fat therefore reduces the downstream influence on the pancreas, increasing the ability of the body to reduce pancreatic fat storage. Reduced pancreatic fat is a key outcome in relation to blood glucose control, as it enables the specialised function of the beta-cells to return for many individuals (71). It is worth noting however that these specialist functions, such as stimulating the first phase insulin response, may be less important in individuals who have reduced their carbohydrate intake; as the inability of the body to effectively deal with dietary carbohydrates is not as relevant to blood glucose control if there is less dietary carbohydrate to be processed within the body.
- **Allowing the pancreatic beta-cells to rest—**Lowering dietary carbohydrate intake reduces the need to shuttle glucose into the cells, thus there is a decreased requirement for insulin. As a result, the workload of the pancreas (for this function at least) is not as high when an individual adopts a LCD. This period of rest may contribute to the return of beta-cell function (63, 72), though further research is required to confirm this.
- **Reduced glucotoxicity—**Insulin production and secretion are negatively affected by glucotoxicity (72, 73), when supraphysiological exposure to glucose over an extended time period causes beta-cell damage (74). Reducing the exposure of the beta-cells to glucose by limiting the intake of dietary carbohydrate may therefore be beneficial (63).
- **Improved blood pressure—**Weight loss is one means through which a LCD may help to reduce blood pressure, though a recent paper assessing the impact of carbohydrate restriction concluded that weight loss alone would could not explain the drop in blood pressure that was observed (75). It is possible that a reduction in insulin levels may play a role in this (75), because insulin causes sodium to be retained in the body (76, 77) which can lead to an increase in blood pressure (78). A LCD also typically results in a reduced intake of highly processed foods, which tend to have a high salt content. As a result, LCDs often lead to both a reduced intake *and* a reduced retention of sodium; and it can even be necessary for individuals following this approach to add salt to their food to prevent sodium levels, and blood pressure, dropping too low (79, 80). As with glucose lowering medications, hypertensive medications should be adjusted at the onset of a LCD (79, 80). This further supports the assertion that LCDs can reduce blood pressure rapidly and, at least in part, independent of weight loss.
- **Reduced triglyceride levels—**LCDs consistently lead to a reduction in triglyceride levels (81), an effect that is likely linked to the ability of this approach to reduce hepatic fat. This is because when hepatic fat is elevated excess triglycerides are shunted into the blood (1). Reducing triglyceride levels can lead to additional benefits, as the amount of triglyceride in the blood has an impact on the size, structure and number of circulating lipoproteins (81). Improvements in these markers result in an overall reduction in cardiovascular disease risk (82), something that is explored further later in the review.

## Existing Evidence

### Limitations in the Body of Research

Before discussing the findings of relevant research in relation to LCDs and Type 2 diabetes further, it is important to consider the limitations of such studies and reviews. This provides important context for the evaluation of the available evidence, particularly as there are a number of limitations commonly seen in work in this field. Consistent issues include:

- the grouping of papers based on target dietary intake rather than actual dietary intake, thus studies are often presented as assessments of low or very low carbohydrate diets despite actual carbohydrate intake exceeding stated thresholds. Demonstrating this, there is as little as 8 g difference in the mean reported carbohydrate consumption of the “high” and “low” carbohydrate groups in some studies (83). Further, in one identified study the carbohydrate intake was actually lower in the control group than the low carbohydrate group at certain time points (84).
- failure to consider the quality of the foods consumed as part of the intervention and/or control diets.
- failure to consider the possible influence of other differences between diets. Differences in protein intake in particular may impact the outcomes of studies in this area, with previous research demonstrating an influence of protein intake on blood glucose control (85–87); whilst differences in meal patterns or eating frequency may also have an effect (88, 89).
- a failure to measure or consider the pre-study dietary intake of the participants, and/or differences between studies in relation to other baseline characteristics (such as duration of diabetes, baseline HbA1c, or insulin sensitivity).
- differences in the level of support provided to the intervention and control arms (i.e., intervention intensity).
- the dietary interventions in some studies are designed to provide an equal amount of energy (calories) in both the LCD and control groups. This precludes any difference being observed as a result of changes in *ad libitum* food intake. Further, differing instructions on this front between groups *within* studies (for example, where one group is instructed to consume food *ad libitum* whilst the other is provided with a strict caloric target) may further influence the outcomes, independently of the influence of the different eating patterns themselves. The possibility that caloric matching, or lack thereof, may modify outcomes was however assessed in a recent meta-analysis, with the authors concluding that there was no evidence that this was the case (90). They did however acknowledge that measurement error in assessment of dietary intake limits the ability to assess this fully, particularly as blinding to dietary allocation is not usually feasible.
- the methods of tracking and/or assessing the diets of the participants are often flawed, an issue that is largely unavoidable but provides a limitation for much of the body of nutrition research. The limitations of dietary assessment methods such as food frequency questionnaires and 24 h dietary recall are well-known, whilst more robust methods such as weighing foods and completing food diaries are still not without flaws and are often not adhered to.
- For logistical and financial reasons it is impractical, and often unfeasible, to perform highly controlled long-term studies. There is therefore a reliance on extrapolating surrogate markers from short-term studies to estimate long-term effects, an issue that is particularly problematic in relation to LCDs as some people require an adaptation period when adopting dietary approaches of this nature before any beneficial effects are apparent (80).
- a failure to fully consider the influence of the intervention and control arms on medication requirements (particularly on anti-hyperglycaemic medication requirements).

### Systematic Reviews and Meta-Analyses

There are a number of published systematic reviews considering the effect of LCDs on weight loss and other markers of health in people with Type 2 diabetes. All identified reviews which included meta-analyses of randomised controlled trials (RCTs) are outlined in Supplementary Table 1 [*n* = 12 (37, 90–100)]. The general conclusion of many of these is that LCDs perform better for weight loss and improving diabetes control in the short-term, but over the longer-term (generally more than 6 months) there is often little difference between LCDs and control arms (which are usually based on low fat diets).

The apparently diminishing differences may be as a result of reduced adherence to LCDs over time (and it is the position of many experts in the field that this is the primary explanation in most cases), may be an artefact of limitations of the reviews (or the studies they include), or indeed they may be a true reflection of the effectiveness of such diets. It is however difficult to fully elucidate the cause(s) based on the available information. One of the included meta-analyses did attempt to assess this, finding, for example, that adherence did seem to explain the apparent reduction in efficacy of VLCDs for weight loss at 6 months (90). The authors of this review also ultimately concluded that it is difficult to determine with any certainty why the effect of LCDs appear to reduce over time though.

Regardless of the reasons for any benefits not being maintained (based on the evidence available in these reviews), these outcomes provide clear and consistent evidence that LCDs can be at least as effective as other dietary approaches. The most appropriate manner within which to consider this evidence is on a non-inferiority basis, as the comparison made in the majority of studies assessing the impact of LCDs is against the existing standard of care. As these findings support the non-inferiority of LCDs they therefore support the use of LCDs as a suitable option in people with Type 2 diabetes.

**Figure 3:**
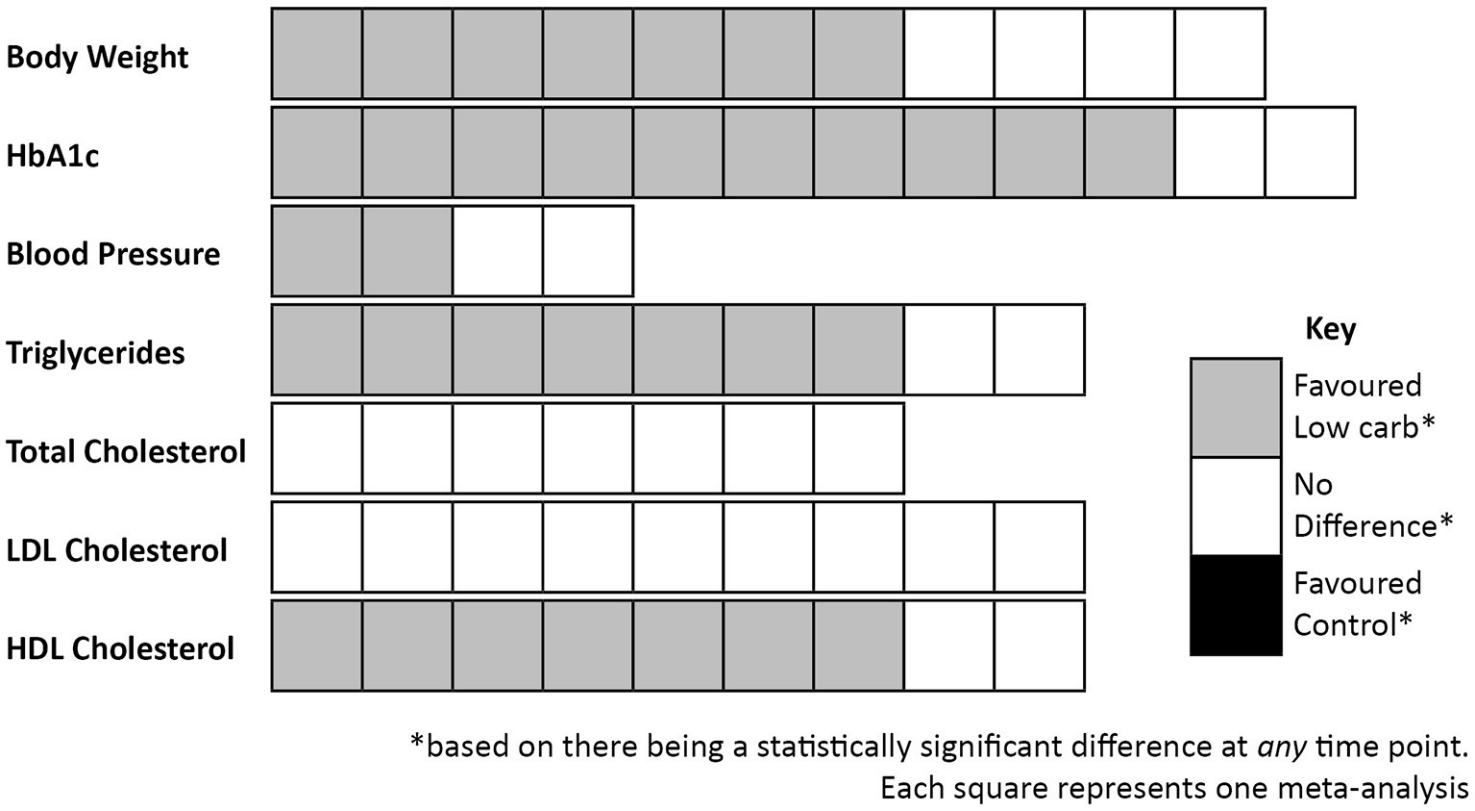
Summary of outcomes from meta-analyses that compared low carbohydrate dietary approaches with control diets in people with Type 2 diabetes.

Notably, where there are differences between groups they are consistently in favour of LCDs (see Table 1). Differences were most commonly seen for body weight (37, 90, 94, 95, 97, 98, 100), HbA1c (37, 90, 92, 94–100), triglycerides (37, 90, 91, 94, 96, 98, 99), and high-density lipoprotein (HDL) cholesterol (90–92, 94, 96, 98, 99); and no statistically significant differences in favour of low fat dietary approaches were observed in any of the meta-analyses for any variable at any time point. It is also worth highlighting that there were no between-group differences in the change in total cholesterol or low-density lipoprotein (LDL) cholesterol in these meta-analyses, both markers which it is often claimed will be affected negatively by a LCD [one review did report that there was a difference between groups for LDL cholesterol (90), but on further assessment this was non-significant]. These outcomes are summarised in Figure 3.

**Table 1:** Summary of significant differences in meta-analyses assessing low carbohydrate dietary approaches for the management of Type 2 diabetes.

	**Body weight**	**HbA1c**	**Blood pressure**	**Triglycerides**	**Total cholesterol**	**Low-density lipoprotein cholesterol**	**High-density lipoprotein cholesterol**
Kodama et al. (91)		–		LCD	–	–	LCD
Ajala et al. (92)	–	LCD		–		–	LCD
Naude et al. (93)	–	–	–	–	–	–	–
Fan et al. (94)	LCD	LCD		LCD	–	–	LCD
Snorgaard et al. (95)	LCD	LCD					
Korsmo-Haugen et al. (37)	LCD	LCD	–	LCD	–	–	–
Huntriss et al. (99)	–	LCD	LCD^*^	LCD	–	–	LCD
Sainsbury et al. (97)	LCD	LCD					
Meng et al. (96)	–	LCD		LCD	–	–	LCD
van Zuuren et al. (98)	LCD	LCD	LCD^**^	LCD		–	LCD
McArdle et al. (100)	LCD	LCD	^***^	^***^	^***^	^***^	^***^
Goldenberg et al. (90)	LCD	LCD		LCD	–	–	LCD

*Articles are listed in chronological order based on the latest date for which they included studies*.

*Table demonstrates where there were statistically significant differences between diets at at least one of the time points included in meta-analyses. No implication is made regarding the clinical meaningfulness of any differences*.

*LCD, favours low carbohydrate dietary approach; –, no difference between diets; grey, not included/reported. No analyses favoured the control diet (usually a low fat diet)*.

* *For systolic but not diastolic*,

** *for diastolic but not systolic*,

*** *Variable included in systematic review, but meta-analyses were not performed*.

Of the main limitations of research in this area, the failure to consider the varying effect of different dietary approaches on medication requirements is a particularly important one. This is because studies consistently demonstrate greater reductions in diabetes medication requirements in the lower carbohydrate arms (see Supplementary Table 1). Where an outcome such as HbA1c is similar between two groups, but one group has achieved this whilst significantly reducing the amount of anti-hyperglycaemic medication they require, this demonstrates a superior performance from the intervention that allowed this. The ability to reduce medication requirements is a strong motivator for many individuals too, and indeed many diabetes medications are reduced or removed at the onset of a LCD (11). Therefore, the failure of many of the reviews to effectively consider this likely penalises the low carbohydrate groups. However, as studies do not report medication usage or changes in a uniform way it is often not possible to pool outcomes related to this. Where medication changes were discussed in the meta-analyses identified for inclusion in the current review the following was reported:

- Snorgaard et al. (95) found that, in the seven studies they included which reported relevant outcomes, medication was reduced at 3 and 6 months with lower carbohydrate dietary approaches compared to higher carbohydrate dietary approaches; and was “numerically lower” at 12 months. The authors acknowledged that “changes in glucose lowering medication have probably led to an underestimation of the effect of low carb diets on glycaemic control.”
- Sainsbury et al. (97) found that, in the 12 studies they included which reported relevant outcomes, there was a greater reduction in medication use for participants on carbohydrate-restricted diets compared with higher carbohydrate diets at every time point; with all studies that reported on such outcomes observing either reduced dosage of oral medications and/or insulin, or an elimination of medication altogether, in the lower carb groups.
- van Zuuren et al. (98) reported that “in all of the studies that included patients taking medication and that adequately reported eventual adaptations, with the exception of one, glucose lowering drug doses were reduced in participants who consumed low-carbohydrate food, but not in those consuming low-fat food.” It should be noted however that this was based on just four studies.
- Korsmo-Haugen et al. (37) concluded that the information available suggests that there was a greater reduction in the use of diabetes medication, particularly insulin, in the LCD groups—and that this may have masked a more positive influence of LCDs on glycaemic control. They acknowledged that this conclusion was based on limited information however, with only four studies showing a significant difference in the change in medications between diets (of the studies they included, it was unclear how many performed analyses to assess whether there was a significant difference for this).
- Huntriss et al. (99) found that, in the 14 studies they included which reported relevant outcomes, every study reported a greater reduction in the requirement for diabetes medication in the low carbohydrate group than in the control group. Of the 11 studies that ran relevant analyses, nine (82%) found this difference to be statistically significant.
- Goldenberg et al. (90) included reduction of medication as a secondary outcome, reporting that LCDs led to greater reductions in the need for diabetes medication at 6 months (risk difference = 0.24, 95% confidence intervals 0.12 to 0.35, GRADE certainty of evidence = moderate) and at 12 months (risk difference = 0.33, 95% confidence intervals 0.00 to 0.66, GRADE certainty of evidence = low). Limited details were presented in relation to these analyses however; for example, which studies were included in the relevant analyses was not reported, and neither was heterogeneity. This factor was still not considered in relation to the assessment of other outcomes either, though the authors did acknowledge that “reductions in medications may blunt the effect on mean HbA1c levels, biassing results toward the null and masking any effect” in their limitations section. The authors also asserted that the ability to assess medication change was impeded by the fact a number of trials placed limitations on what changes to medication were allowed. The degree to which this would have influenced the outcomes is unclear, but based on the other available evidence, as well as the physiology underpinning the requirement for medication reductions, it is likely this would have further biassed them against LCDs.

When appraising this evidence it should be acknowledged that there is an overlap in the studies included within these reviews. They therefore should not each be considered to provide additional, independent evidence. Nevertheless, the findings are clear and consistent.

One final outcome assessed by the most recent of these meta-analyses (90), but not the earlier ones, was remission of Type 2 diabetes. Within this paper it was concluded that LCDs increased rates of remission at 6 months, by 32% when the definition of remission did not require diabetes medication to be omitted, or by 5% if the absence of diabetes medication was required (though the finding was non-significant in the latter case). Remission rates at 6 months were lower in studies which included patients who had been prescribed insulin. At 12 months there was no difference between groups, reflecting the diminishing returns seen for other outcomes. However, in addition to the general limitations outlined previously, this analysis was subject to some additional flaws. For example, the definition used for remission did not include a temporal component, whereas most definitions of remission require at least two measurements to be made, with a minimum duration of time between them (usually 6 months) [e.g., (101)]. The majority of studies from which data were drawn for the relevant analyses did not include remission as an outcome either, thus the use of their findings to draw conclusions on this may be limited.

### Randomised Controlled Trials

As well as considering the conclusions of systematic reviews and meta-analyses it is beneficial to consider the outcomes of individual studies, as this allows the evidence to be appraised in a manner that takes into account some of the limitations identified with existing systematic reviews. For example, it allows a focus on studies where the reported intake of carbohydrate (rather than the stated target intake, which is often greatly different to the dietary intake recorded) was consistent with common definitions of LCDs, and it provides an opportunity to further consider the influence of changes in medication requirements.

For the purpose of the current review, all studies included within the meta-analyses listed in the previous section were considered. To reduce the risk of bias, no additional studies were sought or included in the appraisal of relevant trials reported here. After excluding studies classified as using “moderate” carbohydrate diets in their intervention arm, 71 studies were included from these meta-analyses. This list was then filtered using the criteria applied by the National Institute of Health and Care Excellence (NICE) in their development of the guidelines for the management of Type 2 diabetes in 2009 (subsequent updates to this guideline did not appraise the relevant evidence pertaining to the effect of different dietary approaches). These criteria, which were simply that studies had to have a minimum of 50 participants and a follow up of at least 3 months, were applied so that the outcomes of this assessment could be used to consider how the existing evidence may affect current guidelines in the UK if they were to be updated now. Of the 71 studies identified, only 31 had a stated target for carbohydrate intake in the low carbohydrate arm that was less than the definitions set out at the start of this review (i.e., a carbohydrate intake of <130 g per day). Of these:

- 15 studies had <50 participants (102–116). A number of these references would not have met the inclusion criteria, or at least there would have been question marks over the validity of their inclusion, even if they had a sufficient number of participants. Reasons for this include that some were not published trials [one was a doctoral thesis (106) and one a poster abstract (112), for example] or that there were other significant factors being studied alongside the dietary intervention [for example, in one study the intervention group also took part in an interval training programme (113), whilst in another the control group were prescribed orlistat (107)].
- two studies were not truly studies of LCDs, with one using protein shakes as meal replacements (117) and another assessing “low-carbohydrate and protein sparing modified fasts” (118).
- in one study the reported carbohydrate intake was higher in the LCD arm than in the control group of the study at multiple time points, with the paper also stating that “macronutrient intake did not differ significantly between groups at any point” (84).
- one study was only published as a poster abstract (119).
- two studies had a reported carbohydrate intake above the threshold to be defined as a LCD, despite the target carbohydrate intake meeting the applied criteria (120, 121).

Of the remaining ten publications (21, 22, 24, 122–128) there were three occasions where multiple papers were based on the same trial; reporting different outcomes (124, 125) or different lengths of follow up (21, 122, 126–128). In all cases where multiple publications representing the same trial were identified, the publication with the longest follow up was included. The representative papers of each of the six identified eligible studies are summarised in Supplementary Table 2.

Although a simple means of outlining the findings of these studies, it is of note that the HbA1c reduction was greater in the low carbohydrate arm of all six of the identified trials (21, 22, 24, 123, 124, 128); though the difference between groups was only statistically significant in one (22). For other health markers, most studies demonstrated comparable improvements for LCD and control arms, though where there were statistically significant improvements they were consistently in favour of the LCD arm (see Table 2). Importantly, all five studies that reported relevant outcomes demonstrated a greater reduction in diabetes medication requirements in participants randomised to follow a LCD (22, 24, 123, 124, 128). As noted before, this results in an underestimation of the benefits of LCDs. These findings, based only on studies where reported carbohydrate intake was below the threshold used to define a LCD, support that LCDs are at least as effective as the low fat dietary approaches which have generally been recommended for the management of Type 2 diabetes.

**Table 2:** Summary of significant differences in randomised controlled trials comparing low carbohydrate dietary approaches (carbohydrate intake below 130 g/day or 26% total energy) with any control diet in people with Type 2 diabetes (minimum 50 participants and 3 months duration).

	**Body weight**	**Blood glucose control**	**Blood pressure**	**Triglycerides**	**Total cholesterol**	**Low density lipoprotein cholesterol**	**High density lipoprotein cholesterol**	**Medication change**
Stern et al. (21)	^*^	–	^*^	^*^	^*^	^*^	^*^	
Daly et al. (123)	LCD	–	–	–				LCD^**^
Westman et al. (22)	LCD	LCD	–	–	–	–	LCD	LCD^**^
Goldstein et al. (24)	–	–	–	–	–		–	LCD^**^
Guldbrand et al. (124)	–	–	–	–	–	–	–	LCD
Tay et al. (128)	–	–	–	LCD	–	–	LCD	LCD^**^

*Table demonstrates where there were statistically significant differences between diets at at least one of the time points included in the study. No implication is made regarding the clinical meaningfulness of any differences*.

*LCD, favours low carbohydrate dietary approach; –, no difference between diets; grey, not included/reported. No analyses favoured the control diet (usually a low fat diet)*.

* *Variable included in study, though results not reported independently for participants with Type 2 diabetes*.

** *Statistical analyses not performed for these variables within these studies, though numerical differences were apparent*.

When comparing these studies (see Supplementary Table 2) the evidence supports the previous assertion that a LCD results in a reduced energy intake, a finding that may help to explain the generally favourable health improvements in those randomised to follow LCDs. Three of the included RCTs provided specific guidance to restrict energy intake to the control group whilst allowing *ad libitum* consumption in the LCD (21, 22, 24), yet, despite this, the reported energy intake was lower in the LCD for two of them (21, 24). The third of these RCTs reported a higher energy intake in the LCD. However, the authors acknowledge that this may reflect limitations in the data collection rather than being a true finding (22), particularly as weight loss was significantly greater in the LCD group, though they do also suggest that this difference in weight loss could be as a result of increased energy expenditure following a LCD (thus may be a true finding); an assertion that has some support from other experimental data (26, 42). Of the other RCTs, one did not provide a target for energy intake to either group (with the reported energy intake subsequently being lower in the LCD arm) (123) and the other two provided guidance aiming to ensure the two dietary arms were isocaloric (124, 128). In these latter two studies, there was no difference in the reported energy intake of the two groups for one (128), though in the other the reported energy intake was still lower for the LCD arm (124).

As introduced earlier, protein intake is another factor that may influence outcomes in studies of this nature (85–87). Three of the identified studies did not provide specific protein targets to either group (21, 22, 123) [though the dietary guidance provided to the control group appeared to be more prescriptive in one of these studies (22), thus would likely have influenced protein intake], one provided a target to the control group but not the LCD group (24), and two provided a target to both groups (with a higher target as a percentage of total energy to the LCD arm in both cases) (124, 128). Across all of these studies intake was generally similar between groups (21, 124), or was slightly higher in the LCD arm (22, 24, 123, 128). However, of the studies reporting a higher protein intake in the LCD group the difference in one was only apparent when considered as a percentage of total energy intake, with the mean absolute intake (as calculated by the authors of the current review) being just ~10 g/day different between groups (123). The possible limitations in dietary assessment in another have already been noted (22). There therefore appears to be little difference in the absolute protein intake between groups, regardless of whether participants were provided with targets or consumed food *ad libitum*, despite consistently lower energy intakes in the LCD arms. This supports the important role of protein in relation to satiety [in line with the predictions of the protein leverage hypothesis (47, 48)], and suggests that the foods included in the LCD arms of these studies had a higher proportion of protein than those in the comparison arms (i.e., the total amount of food required to reach this protein intake was less in the LCD groups); which may have had an influence on the outcomes.

When considering the evidence pertaining to carbohydrate restriction it is sometimes argued that low and very low energy diets should be included, as such interventions often meet the stated criteria in relation to absolute carbohydrate intake. Indeed it is possible that at least part of the benefit seen from interventions of this nature, for example DiRECT (71, 129), could be attributable to carbohydrate restriction rather than being solely due to low energy intake or weight loss. One mechanism through which this may be the case is by allowing the beta-cells time to rest and recover, which energy restriction in the absence of carbohydrate restriction would not allow to the same degree. This could conceivably contribute to the re-differentiation of the beta-cells, a key feature in the remission of Type 2 diabetes (71). The potential benefits of nutritional ketosis, for example in relation to reducing hunger, have also already been introduced. However, these approaches are not designed to be long-term and do not represent LCDs in a form congruent to that which would usually be promoted. Therefore, evidence of this nature is not considered further in the current review.

### Other Sources

RCTs and systematic reviews/meta-analyses of such trials are considered to be the highest quality of evidence, but important information can also be obtained from alternative sources. It is also important to acknowledge that some questions are better answered by alternative forms of research, and that the overall quality of a study, including the appropriateness of the analytical methods, is often a more important consideration than the type of research that has been performed. Further, although other forms of evidence may be considered inferior based on some criteria, they can also have their own strengths, for example they are often more ecologically valid. This type of evidence can therefore help to bridge the gap between research and practise.

One key example is the work of Virta Health, who have demonstrated outcomes that support the safety and efficacy of carbohydrate restriction for the management, and possible remission, of Type 2 diabetes (38, 130). The aim of the intervention used was to achieve a sustained state of nutritional ketosis, which required a carbohydrate consumption of <30 g per day (a VLCD) for most of the participants. After two years, 55% of those following the VLCD had an HbA1c that would be classified as non-diabetic, 67% of all diabetes medications used at baseline were no longer required, mean insulin dose was reduced by 81% (from 81.9 units/day to 15.5), and over 60% of those using insulin were able to omit it altogether (38). Virta Health have also demonstrated meaningful reductions in cardiovascular disease risk in participants of their programme, with a mean reduction in 10 year atherosclerotic cardiovascular disease risk score of 11.9% (82). Their 2 year results showed an average reduction in triglycerides (−22%) and CRP (−37%), and the resolution of metabolic syndrome in 29% of participants (38); whilst additional analyses of 2 year outcomes found a decrease in the number of small LDL particles and that there had been no progression of carotid intima media thickness (131). The primary limitation of this evidence is that participants were not randomised to the intervention or control group, i.e., those who followed the VLCD chose to do so. However, as alluded to previously, the results are therefore perhaps a better reflection of real world effectiveness, demonstrating that a self-motivated group of individuals who have made an informed decision to follow a VLCD can adhere to this eating pattern and achieve clinically meaningful results through doing so.

Additional evidence that lacks the methodological rigour of a controlled trial but provides a useful insight into the effectiveness of LCDs in a free-living situation is provided by the Diabetes.co.uk Low Carb Programme. Outcomes from a sample of 1,000 participants of this programme, randomly selected from a larger convenience sample, have been published (132); with the primary limitations of this evidence from a research perspective being that there was no control group and that the results were self-reported. The results nevertheless provide support for the effectiveness of LCDs, with 195 (26.2%) of the 743 participants who had an HbA1c above the threshold for Type 2 diabetes diagnosis at baseline reducing it to below this threshold after 12 months. Further, 46.4% of participants lost at least 5% of their initial body weight; and of the 714 participants who were taking at least one medication to manage their diabetes, 289 (40.4%) reduced one or more of these medications. Notably, this programme has now had over 400,000 users; demonstrating the increasing popularity of LCDs.

Evidence of the safety and effectiveness of LCDs for the management of Type 2 diabetes has also been shown within primary care in the UK (75, 133–135). For example, drug-free remission of Type 2 diabetes [defined as achieving an HbA1c of <48 mmol/mol (6.5%)] has been recorded in 56% (68/149) of the patients who have adopted a LCD in one practise (135). The LCD intervention delivered at this practise has also demonstrated considerable financial benefits, with the overall diabetes drug spend previously reported as being ~£45,000 less per year than the regional average (136); a saving that has now increased to >£50,000 per annum despite an increase in the unit cost of many of the medications in question (135).

A further investigation of the impact of a LCD in a community-based setting in the US provides additional support for its effectiveness, finding significant improvements in HbA1c and body weight in patients with Type 2 diabetes who opted for this approach (137). Patients in the LCD group showed a reduction in HbA1c that was 1.29% greater than that observed in the usual care control group (95% CI: −1.75% to −0.82%, *P* <0.001), whilst weight loss was 12.8 kg more in the LCD group (95% CI: −14.7 kg to −10.8 kg, *P* < 0.001). All patients in the LCD group who were taking insulin at baseline were able to reduce or discontinue this therapy (compared with 23.1% in the control group), and there were no safety issues reported. Adherence to a LCD, defined as <20 g carbohydrate per day, was reported to be high, with detailed food logs used to verify this.

## Inclusion of Low Carbohydrate Dietary Approaches in National and International Guidelines

Arguably above and beyond the quantity and quality of available evidence for a particular approach or intervention, the position of bodies who produce relevant policies and guidelines is highly significant. This is because many healthcare professionals, particularly those who are not specialists within a given area, are not familiar with emerging evidence, and/or do not have the time or skills to fully appraise it. As a result they will defer to the position of relevant organisations to guide their practise, and will often not feel comfortable or confident with promoting and supporting an approach until they perceive it to be supported by such bodies. Importantly, a number of influential organisations now support the use of LCDs for the management of Type 2 diabetes:

- Diabetes UK (DUK) guidance from 2011 (138) and 2018 (139) concluded that there is insufficient evidence to promote a specific dietary approach or to conclude what percentage of a person's energy intake should come from fat, protein, or carbohydrate. They state that adherence to a dietary approach is the best predictor of long-term success, and that individualisation of approaches is important. They support carbohydrate restriction as a suitable option as part of this. A 2017 DUK position statement (140) also supports the use of LCDs, with caveats regarding uncertainty over the longer-term effect of such approaches (a common concern that is discussed later in the current review).
- The British Dietetic Association (BDA) released a position statement in 2018 supporting carbohydrate restriction as a viable option for adults with Type 2 diabetes (141), with caveats around the need for more research to ascertain the ideal nutritional composition and the long-term effects.
- The Scottish Intercollegiate Guidelines Network (SIGN) updated their national clinical guidelines for the management of diabetes in 2017 (142). These guidelines recommend that people with Type 2 diabetes be given dietary choices for achieving weight loss that may also improve glycaemic control. The listed options for achieving this include restricting the total amount of carbohydrate that is consumed, though they include caveats regarding the degree of restriction (recommending a minimum of 50 g carbohydrate per day) and duration (stating that this appears to be safe for up to 6 months).
- A 2018 joint position statement from the ADA and the European Association for the Study of Diabetes (EASD) reached conclusions similar to DUK, promoting individualised dietary approaches for patients, with LCDs being listed as a suitable option (143).
- A 2019 consensus report from the ADA concluded that “a variety of eating patterns are acceptable for the management of diabetes” supporting the need to individualise approaches (10). In relation to carbohydrate restriction specifically, this report acknowledges that “Reducing overall carbohydrate intake for individuals with diabetes has demonstrated the most evidence for improving glycaemia and may be applied in a variety of eating patterns that meet individual needs and preferences”; “For select adults with type 2 diabetes not meeting glycaemic targets or where anti-glycemic medications is a priority, reducing overall carbohydrate intake with low- or very low-carbohydrate eating plans is a viable approach”; and “…from the current evidence, this eating pattern does not appear to increase overall cardiovascular risk….”. The position set out in this consensus report was subsequently included in the 2020 update to the ADA Standards of Medical Care in Diabetes (144).

However, the current guidance provided by the National Institute of Health and Care Excellence (NICE) in the UK is not fully consistent with the organisations referenced above (145). Although elements of this guidance are aligned with these other bodies, for example it includes recommendations to favour lower glycaemic index carbohydrates and to individualise carbohydrate intake and meal timings, the overriding message is to promote the same way of eating that is recommended for the general population [i.e., The Eatwell Guide (146) in the UK, which is based on low fat principles]. When updating these guidelines in 2019 it was decided that the section on dietary approaches did not warrant review. This is despite multiple stakeholders (including DUK, the BDA, and several of the authors of the current review on behalf of X-PERT Health) challenging this position during the consultancy phase. Inconsistency in guidance has the potential to cause confusion. This was therefore a missed opportunity to help healthcare professionals, who, in the UK, are more likely to defer to NICE guidance where there is any doubt over what constitutes best practise in an area they do not specialise in, feel comfortable with supporting their patients to adopt LCDs when they are interested in doing so, in line with the position of multiple other relevant organisations (as set out previously). Of note though, the overall response summarising decisions made pertaining to the update of this guideline did state “NICE guideline NG28 already advises individualising recommendations for carbohydrate intake, and meal patterns, which could include low carbohydrate and low calorie diets” (147). It is therefore clear that the promotion and support of LCDs for people with Type 2 diabetes is not precluded by the NICE guidelines in their current form.

## Areas of Controversy

### Long-Term Effects of Low Carbohydrate Dietary Approaches

Although the positions outlined in the previous section acknowledge the potential utility of carbohydrate restriction for people with Type 2 diabetes, there are still caveats regarding the longer-term effects of this approach in many of them. It could be argued however that these qualifying statements, which are generally not applied to other patterns of eating, are not supported by the available evidence.

The first argument against the necessity for such caveats, particularly when they are not used for other ways of eating, it that there is an absence of high quality evidence regarding the long-term safety and efficacy of ANY dietary approach. This is perhaps largely due to the limitations of nutritional research, some of which were outlined earlier within this review. These limitations are true in relation to the study of all dietary approaches, including low fat approaches which have been adopted widely by organisations worldwide and Mediterranean-style approaches which are almost universally accepted as healthy. Where longer-term studies have been attempted, the outcomes in relation to low fat dietary approaches, for example, often do not support its superiority over other approaches. For example, in the Women's Health Initiative study glycaemic control was worse in the low fat arm than the control group after 6 years (148) and in the LookAHEAD trial there was no reduction in cardiovascular disease risk in the intervention group when compared to the control group, with the study being stopped after 9.6 years as a result (149). This raises questions as to why low fat approaches are widely promoted without qualification, yet LCDs are promoted with caution due to a perceived absence of long-term evidence. It is inappropriate to hold LCDs to a level of evidence higher than that which other dietary approaches can meet.

Much of the research used to show associations between LCDs and adverse outcomes, evidence often cited in opposition to the use of LCDs, is of an epidemiological nature. However, the individuals classified as following a LCD within these studies are invariably not people who have made a conscious decision to restrict carbohydrate intake in a manner, or to the degree, that would be considered a true LCD. They therefore cannot be considered to be representative of this population, or, by extension, the long-term impact of carbohydrate restriction. One clear example of inappropriate classifications of diet for this purpose is provided by a recent study which classified people as following a LCD if <50% of the energy they derived from food was from carbohydrate (and more than 35% was from fat) (150). When split into deciles, the upper limit for carbohydrate intake in the *lowest* carbohydrate group of this study was 39.5% energy from carbohydrates; significantly above the standard definitions outlined earlier within this review. Beyond the other limitations of observational research, which would preclude drawing definitive conclusions regardless, this clearly does not provide a valid representation of a LCD. This study is indicative of the methods used in much of the comparable research [e.g., (151–154)].

One limitation of epidemiological evidence that may be particularly relevant when assessing LCDs is the possibility of healthy/unhealthy user bias, an issue which could influence the results in either direction. Some individuals who ignore existing dietary guidelines and reduce dietary carbohydrate intake may also ignore other guidelines and exhibit behaviours known to have a negative influence on health, such as smoking or being inactive (unhealthy user bias). Conversely, individuals who opt to reduce carbohydrate intake may be doing so as a result of being aware of the potential benefits, and thus may be more health conscious than the general population. This makes them more likely to adopt other behaviours that may be health promoting (healthy user bias).

The confounding influence of alcohol intake is also potentially significant. Alcohol consumption is poorly recorded through observational data collection methods, with intake commonly being underreported (particularly in people who consume excessive quantities of it) (155). Attempts to control for alcohol consumption are therefore severely limited. Furthermore, carbohydrate is the macronutrient that is most commonly displaced by alcohol in individuals who consume it to excess (156). There is therefore a possibility that a lower carbohydrate intake may be reflective of alcoholism (or, at least, of an elevated intake of alcohol, which may affect health detrimentally through both direct and indirect effects) in some people, rather than being due to a deliberate adherence to a LCD in a manner consistent with that discussed in the current review. The inability to fully account for this when analysing epidemiological data, and the consistent failure to consider it when discussing the findings, is a major limitation of this body of research.

A further argument against the use of qualifiers relating to long-term effects when recommending LCDs is that the evidence that does exist does not support the contention that this approach increases cardiovascular disease risk, the issue which is usually the central tenet of such concerns. In the absence of high quality, long-term evidence assessing differences in disease prevalence, cardiovascular mortality and/or total mortality between different dietary approaches, studies considering changes in relevant risk markers provide the best available evidence. Although there are clear limitations with this, changes in cardiovascular markers for the LCD arms of such studies are comparable, or often favourable, to those of the control groups; which are usually based around low fat dietary approaches. In support of this assertion, and as noted previously, the ADA 2019 consensus statement on nutrition therapy for adults with Type 2 diabetes concluded that, based on the available evidence, there was no increase in overall cardiovascular risk in individuals who followed a LCD, and, notably, that this appears to be true even within studies where the intake of saturated fat was increased (10). Furthermore, none of the meta-analyses identified for inclusion in the current review found statistically significant differences in the change in total cholesterol or LDL cholesterol between LCDs and control groups (see Table 1); whilst where there were differences in other markers of cardiovascular disease risk they consistently favoured the lower carbohydrate arms. The same is true for the individual RCTs included (see Table 2), of which several (21, 24, 128) followed participants for 12 months or more; whilst Virta Health also demonstrated a clinically meaningful reduction in cardiovascular disease risk in participants who adopted a VLCD (38, 82, 131).

Despite the absence of evidence that LCDs increase the rate of cardiovascular events, and the observation that, on average, LDL cholesterol does not appear to be increased in individuals with Type 2 diabetes who adopt a LCD, this is still an area of concern for many. There is a question mark however over whether an increase in LDL cholesterol alone should be sufficient to justify the cessation of a LCD in individuals who do experience this change (157). Importantly, the available evidence is clear that the number of LDL or apolipoprotein-b particles is a better indicator of cardiovascular risk than LDL cholesterol is (158, 159), while LDL particle size also appears to play an important role (158). Evidence from LCDs demonstrates that the size of LDL particles tends to increase, whilst the number of smaller particles reduces and the total number of LDL particles either reduces or stays consistent (38, 131). These factors would indicate a reduced (or at least a consistent) level of cardiovascular disease risk. Alongside the improvements regularly seen in other markers of health this usually constitutes a significant overall reduction in cardiovascular disease risk, based on contemporary risk calculators.

Further, it has been posited that changes in LDL in response to dietary changes may not always be pathological. Although further research is required to explore this further, credible hypotheses include that such changes may reflect a necessary adaptation to allow rapid redistribution of cholesterol between specific cells (160), or that they may be caused by an increase in circulating free fatty acids, due to increased lipolysis (facilitated by reduced insulin levels), with an ultimate purpose of providing fuel for the body (161). Both of these examples provide a possible explanation for why an increase in LDL cholesterol may represent a normal physiological process, rather than a pathological change. The presence or absence of other negative health markers, and/or of positive or negative changes in other health markers, should help to provide a clue on the overall metabolic health status of an individual, with clustering of risk factors generally providing greater predictive ability than individual markers. It should also be considered that cholesterol markers may be increased transiently during weight loss, thus clinical assessment may be more appropriate after an element of weight stability has been established.

If LDL elevation remains a concern despite the mitigating factors set out above, it is often possible to achieve a change in them through making changes to the type of dietary fat that is consumed (by swapping some saturated fat for some monounsaturated or polyunsaturated fat) or by increasing dietary fibre intake (by increasing the consumption of green leafy vegetables, for example). Although there may be some debate over the extent to which this is necessary (depending on the context and the overall health status of the individual), such changes may help to provide peace of mind to patients and healthcare professionals alike.

Based on these points, caveats in the position of other organisations pertaining to the long-term effects of LCDs can perhaps be challenged; though it is of course essential that this topic continues to be assessed to increase our understanding and to ensure that there are no negative effects in the longer-term.

### Adherence to Low Carbohydrate Dietary Approaches

Another common criticism of LCDs is that people are not able to continue to follow them long-term, a term which has no universally agreed definition, but is often defined as >12 months in a research setting. Evidence suggests that the approach which is most likely to result in lasting health improvements is whichever one the individual is able to continue following (8), so this is an important issue. This finding also emphasises the important of a range of options being available to patients, to help them find one that is suitable for their personal needs and preferences. However, the available evidence does not support the assertion that LCDs are more difficult to follow than other dietary approaches.

**Figure 4:**
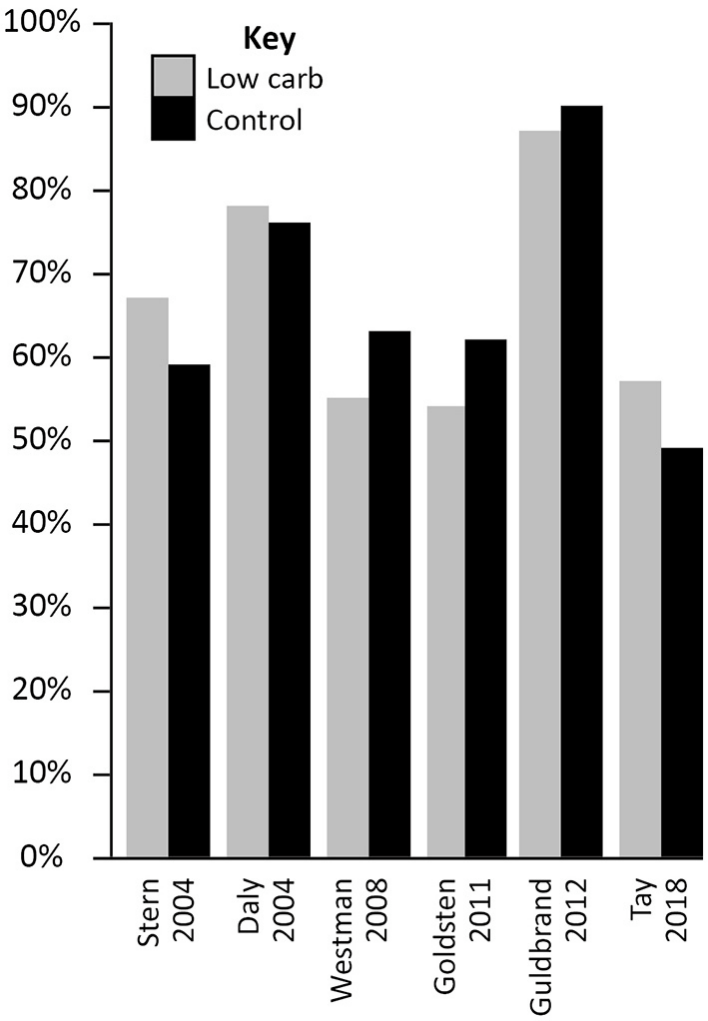
Study completion rates in randomised controlled trials comparing low carbohydrate dietary approaches (carbohydrate intake below 130 g/day or 26% total energy) with any control diet in people with Type 2 diabetes (minimum 50 participants and 3 months duration).

In the Virta Health trial, for example, where participants were highly motivated and self-selected the diet, adherence was 83% at 1 year (130) and 75% at 2 years (38). These values were for a VLCD, which may be more difficult for many people to stick to than more moderate carbohydrate restriction. In RCTs, where individuals may be randomised to an approach that is not consistent with their own preferences, completion rates are similar for LCD and control arms. Indeed, in the six trials included in Table 2 (each of which is summarised in Supplementary Table 2) the average completion rates at the most recent time points for which data are available were 66% in both the intervention and control arms. The completion rates within each study are presented in Figure 4. Although trial completion rates are not a perfect marker of adherence they do provide an indication of this, particularly within studies where reported carbohydrate intake is consistent with the target carbohydrate intake (as it was in the studies included here). It is possible that adherence rates in these studies may be higher than in a general population however. Some studies provide high levels of support, or even provide the participants with the food they are required to consume, both factors which would make sticking to a dietary approach easier than it would be in the real world. Further, many studies are of a short duration, which might help motivate individuals to stick to a LCD even if they are not finding it enjoyable. It is also possible that individuals volunteer for such studies as they have an interest in a LCD, which may result in favourable adherence rates for the LCD arms when compared to the control arms. Supporting this, in one study (which did not meet the criteria for inclusion in the current review as the sample size was below the 50 participants minimum applied) all of the participants who withdrew from the control group did so because they were disappointed at not being randomised to the low carbohydrate arm (104). Despite these possibilities, there is still an absence of evidence that LCDs are more difficult to follow than other ways of eating.

In people's day-to-day lives however, there are still practical barriers to adopting LCDs. These barriers include, in the UK, that reference intakes and traffic light colour coding on food labels are designed to support a low fat dietary approach; that most readily available food in shops and eateries is geared toward a low fat way of eating (for example sandwiches, crisps and chocolate bars are still the most abundant lunch and snack options in most places); and that many people are not supportive of their friends or family members if they try to follow a LCD, due to misconceptions regarding the safety and efficacy of such approaches. These factors may make it more difficult for someone to adopt a LCD, but that does not mean that it is not possible. Indeed, anecdotal evidence supports that many people are able to sustain a LCD long-term. Furthermore, in a survey of dietitians in the UK the majority responded that they felt a LCD was achievable for the “right individuals” as long as they received appropriate support (162). Of note, the majority of respondents to this survey felt that guidance suggesting a 50% energy intake from carbohydrates is inappropriate for people with Type 2 diabetes (162).

Although these barriers may pose challenges to adherence, and may result in some individuals deciding against persisting with a LCD, there is evidence demonstrating that not everybody is put off by such factors. For example, a study reporting findings from interviews with people with Type 1 or Type 2 diabetes who had adopted a VLCD found that participants were sufficiently motivated by the benefits they had seen to persist with this dietary approach despite a perceived lack of support from healthcare professionals (163). Of note, the majority of individuals in this study intimated an intention to maintain this dietary approach for the rest of their lifetime, indicating that they saw it as a lifestyle change rather than a temporary therapeutic intervention.

As LCDs become more widely accepted some of the existing barriers may dissipate, which will help more people to adhere to such dietary approaches. However, changes in the food environment (which are necessary above and beyond the need to make alternative dietary approaches more accessible, with such issues, arguably, being the root of the twin epidemics of obesity and Type 2 diabetes being experienced in much of the Western world, and increasingly beyond) are tied more closely to economic priorities than health-related ones; thus are not likely to occur rapidly. It is therefore crucial that people wishing to adopt a LCD are provided with the education and support necessary to help them make informed decisions about their food choices. Practical advice is available through a number of channels, including group based structured education and digital platforms (including mobile phone applications and websites). Support for healthcare professionals, to help them learn about LCDs and how they can support their patients in adopting them, is also available [e.g., (79, 80)].

### Additional Concerns

Beyond those discussed above, there are a number of other factors often raised as concerns in relation to LCDs. One such concern is that LCDs increase the risk of hypoglycaemia. Although mild hypoglycaemia can be experienced by anyone, severe hypoglycaemia is only a danger for individuals taking certain medications, particularly insulin or sulphonylureas. At the onset of a LCD these medications are reduced or omitted (11), thus there is actually a *reduced* risk of hypoglycaemia in individuals following a LCD, as long as they have a medication review before they begin. Of note, there were no serious adverse events reported in the RCTs identified for inclusion in the current review (see Supplementary Table 2), and where reported in the identified systematic reviews (see Supplementary Table 1) there was no difference in adverse events, including hypoglycaemia, between diets.

An additional criticism sometimes levelled at LCDs is that they increase the risk of nutrient deficiencies. Any dietary approach can result in individuals not obtaining all of the dietary components that the body requires to function optimally, particularly if they are not well-planned or are not based on nutrient dense foods. There is, to the knowledge of the authors, no evidence that LCDs increase the risk of this however. In relation to LCDs concerns are often raised in relation to fibre in particular. However, there are a wide variety of foods that can provide fibre without contributing large quantities of carbohydrate to the diet, such as vegetables, nuts, seeds and dark chocolate; whilst the staple components recommended when individuals adopt LCDs are invariably nutrient dense foods, such as non-starchy vegetables, eggs and oily fish. This is further discussed by Zinn et al. (164), who provide a hypothetical case study demonstrating that LCDs need not be deplete of any important nutrients.

Lastly, it is sometimes asserted that LCDs are more expensive than other ways of eating. Health inequalities are a major issue, with any intervention which is not suitable for individuals with a lower socio-economic status exacerbating the problem. It has been posited that LCDs are not suitable for lower income households, but that is based on false assumptions regarding the types of food an individual may regularly consume when following this approach. As with any dietary approach there are more expensive options which may not be suitable for everyone, but there are also multiple ways a LCD can be adapted to be more cost-effective. For example, staple ingredients such as eggs or tinned oily fish need not be expensive; whilst purchasing tinned or frozen vegetables, for example, can reduce waste and help save money. An analysis of the cost of food for a family of four switching from a low fat diet to a LCD supports that this approach does not need to be significantly more costly (165). It should also be considered, as discussed previously, that LCDs have been demonstrated to reduce hunger for many people. This can lead to a reduced intake of food, including less snacking and/or a lower eating frequency, and thus less money is required to be spent on food. It is important that socio-economic factors are not disregarded though, and people should be supported to adopt the best quality diet that they can afford, regardless of which dietary approach they wish to follow.

## Type 2 Diabetes Remission

The evidence pertaining to Type 2 diabetes remission suggests that LCDs may have an advantage over other dietary approaches. Until recently it was not known or accepted that Type 2 diabetes remission was possible at all (12), suggesting that standard dietary management approaches are not effective for achieving this. Although there is now evidence that Type 2 diabetes remission is possible with a number of approaches [including Mediterranean (166) and low fat diets (167–169)], the evidence is strongest for very low energy diets (129, 170) and VLCDs (38, 130). The former of these methods, very low energy diets, is different in nature to a VLCD however as it is only a short-term approach; thus a long-term, sustainable dietary approach still needs to be adopted after the weight loss phase to ensure any weight that has been lost is not regained, which would almost inevitably lead to the return of Type 2 diabetes. Additionally, the success of a very low energy diet in relation to Type 2 diabetes remission is dependent on the ability of the pancreatic beta-cells to regain their specialist function (71). Evidence suggests however that some people, particularly those who have had Type 2 diabetes for a longer duration (171), may not be able to achieve this. In these cases individuals may not see their ability to effectively metabolise carbohydrates return, and so reducing the amount of dietary carbohydrate they are required to process is likely a better strategy to help them improve their blood glucose control than targeting weight loss alone.

As noted earlier, Type 2 diabetes remission was included as an outcome of interest in a recent meta-analysis (90). This study found differences in remission rates at 6 months that favoured LCDs, though the magnitude of this difference varied depending on the definition of remission used, and appeared to reduce over time. There were however several limitations to this analysis, as discussed earlier, thus alternative sources of evidence may provide more valid assessments of the efficacy of LCDs and VLCDs for helping individuals achieve remission. What is clear from other analyses, including the outcomes of Virta Health (38, 130) and evidence from primary care in the UK (135), is that carbohydrate restriction appears to be a viable option for helping motivated patients to achieve this goal.

## Strengths and Limitations of the Current Review

The primary strength of the current review is that it has considered this topic more broadly than previous analyses have, whilst considering the evidence, from both a clinical and an academic perspective, in the context of its limitations. A number of meta-analyses have been performed in an attempt to provide a definitive answer to the question of whether a LCD is effective for the management of Type 2 diabetes, but as many of these papers are subject to the same limitations they provide limited additive benefit to previous work. A narrative review provides an effective way to address this issue, as a more discursive approach allows an exploration of this information alongside other modes of investigation to draw more meaningful conclusions.

Although the narrative nature of this review can be considered a strength, it can also be considered its main limitation. Compared to a systematic review, a narrative review has an increased risk of bias, and repeatability is reduced as there is no method or search strategy that others can replicate. There is also an increased risk that pertinent evidence may have been missed. However, that part of the review was semi-structured, with all trials included within identified meta-analyses considered, may help to mitigate for this. Another limitation that arises as a direct consequence of one of the papers strengths is that the broad scope of the review necessitates that it has not been possible to explore some important concepts in detail. This includes additional exploration of the potential differences between LCDs and VLCDs, in relation to whether stricter carbohydrate restriction is more effective than more moderate carbohydrate restriction and whether there may be additive benefits due to direct effects of nutritional ketosis. It was not however the purpose of the current review to provide a comprehensive review of all relevant factors, but to provide an update of the state of the existing evidence, and to interpret this in a manner that will help to inform clinical practise and future academic directions.

## Practical Recommendations

### Clinical Practise

- Patients should be supported to make a choice that fits their needs and preferences. There should be less focus on promoting a particular approach as best, and more on allowing individuals to make an informed choice to help them establish which approach is most suitable for them.
- LCDs should be one of the options that are offered, and the possible benefits should be made clear. This should include that existing evidence suggests that LCDs are at least as effective as other approaches for the management of a range of key health markers, and are likely to be superior for outcomes such as a reduced need for diabetes medication.
- To support adherence, and the ability to make informed choices, patients should receive appropriate and ongoing education and support.
- Concerns around the long-term impact of LCDs, or of the ability to adhere to them, should not be used to discourage people from adopting such approaches as they are not supported by the available evidence. However, as with all patients (and all interventions), key health markers should be tracked to allow consideration of the impact of the specific changes made by the individual.
- Where relevant, patients should have a medication review before they begin restricting carbohydrate intake, and relevant health markers should be tracked carefully.
- Individuals with more complex needs, such as the presence of comorbidities and/or with special dietary requirements, should receive additional support from appropriately qualified healthcare professionals when making changes to their diet and lifestyle.

### Research

- Further meta-analyses assessing the impact of LCDs on health should be discouraged, unless they explicitly address the question in a different way (such as stratifying outcomes based on reported carbohydrate intake rather than intended carbohydrate intake).
- Studies should avoid making claims about the impact of LCDs when there is no evidence that those with data included in the study followed a dietary approach that would meet the generally accepted classification of a LCD.
- The possible effects of carbohydrate restriction independent of weight loss should be explored further. It should however be acknowledged that such investigations are primarily academic, and that the mechanism through which an intervention is effective is largely redundant from a clinical perspective.
- The potential impact of differing protein intake and of different types of fat on the safety and efficacy of LCDs, as well as of other potentially influencing factors such as the frequency of eating, should be further explored.
- Future research should ensure that other important factors, such as changes in medication use and the quality of the diet, are fully considered.
- As with all nutrition research and dietary approaches, continued attempts to improve the quality of studies, including through the investigation of objective markers of dietary intake, as well as efforts to improve adherence to dietary changes, would be of great value. This would also help to further assess the long-term impact of different dietary approaches, something that is difficult with existing methods.
- Future reviews should expand on some of the topics that were covered in the current review but, by necessity, were not explored in detail. Consideration of the potential impact of VLCD (i.e., “ketogenic”) approaches should also be explored further, including whether the effects of this are greater than that of more moderate carbohydrate restriction, and an exploration of practical considerations such as adherence and acceptability.

## Conclusion

Available evidence supports the safety and efficacy of LCDs for the management of Type 2 diabetes, with findings consistently demonstrating such approaches to be at least as effective as other ways of eating for improving blood glucose control and reducing cardiovascular disease risk. Further, LCDs appear to be superior to other dietary approaches for reducing the requirement for diabetic medications and, potentially, for placing Type 2 diabetes into remission. Existing evidence does not appear to support the assertion that LCDs are more difficult to adhere to than other dietary approaches. LCDs should therefore be promoted as a possible option for the management of Type 2 diabetes, and where patients make an informed choice to adopt a LCD they should be supported by their healthcare team to help maximise their chances of achieving their health goals.

## Author Contributions

SW completed the initial draft of the manuscript. All other authors contributed to the development of the manuscript.

## Acknowledgements

The authors would like to thank Matthew Whitaker and Nina Evans for providing feedback on an early draft of the current review.

## Conflict of Interest

TD is the founder and chief executive of X-PERT Health. SW, PH, and TR are employees of X-PERT Health. X-PERT Health is a registered charity and not-for-profit organisation which provides structured diabetes education. This education includes information on low carbohydrate dietary approaches, introducing them as a potentially suitable option (alongside other dietary approaches) for people with, or at risk of, Type 2 diabetes. X-PERT Health also produce the *Eat Fat! Handbook*, which provides additional help and support for people who wish to adopt a low carbohydrate dietary approach. Any income from sales is reinvested into research and development. The remaining author declares that the research was conducted in the absence of any commercial or financial relationships that could be construed as a potential conflict of interest.

## References

[1] Feinman RD Pogozelski WK Astrup A Bernstein RK Fine EJ Westman EC. Dietary Carbohydrate restriction as the first approach in diabetes management. Critical review and evidence base. Nutrition. (2015) 31:1–13. 10.1016/j.nut.2014.06.011 25287761

[2] DeFronzo RA Tripathy D. Skeletal muscle insulin resistance is the primary defect in type 2 diabetes. Diabetes Care. (2009) 32(Suppl. 2):S157–63. 10.2337/dc09-S302 19875544 PMC2811436

[3] Roden M Shulman GI. The integrative biology of type 2 diabetes. Nature. (2019) 576:51–60. 10.1038/s41586-019-1797-8 31802013

[4] Rizza RA. Pathogenesis of fasting and postprandial hyperglycemia in type 2 diabetes: implications for therapy. Diabetes. (2010) 59:2697–707. 10.2337/db10-1032 20705776 PMC2963523

[5] Fonseca VA. Defining and characterizing the progression of type 2 diabetes. Diabetes Care. (2009) 32(Suppl. 2):S151–6. 10.2337/dc09-S301 19875543 PMC2811457

[6] Taylor R. Type 2 diabetes: etiology and reversibility. Diabetes Care. (2013) 36:1047–55. 10.2337/dc12-1805 23520370 PMC3609491

[7] Del Prato S Tiengo A. The importance of first-phase insulin secretion: implications for the therapy of type 2 diabetes mellitus. Diabetes Metab Res Rev. (2001) 17:164–74. 10.1002/dmrr.198 11424229

[8] Diabetes UK. Evidence-Based Nutrition Guidelines for the Prevention and Management of Diabetes (2018).

[9] Westman EC Tondt J Maguire E Yancy WS Jr. Implementing a low-carbohydrate, ketogenic diet to manage type 2 diabetes mellitus. Expert Rev Endocrinol Metab. (2018) 13:263–72. 10.1080/17446651.2018.1523713 30289048

[10] Evert AB Dennison M Gardner CD Garvey WT Lau KHK MacLeod J. Nutrition therapy for adults with diabetes or prediabetes: a consensus report. Diabetes Care. (2019) 42:731–54. 10.2337/dci19-0014 31000505 PMC7011201

[11] Murdoch C Unwin D Cavan D Cucuzzella M Patel M. Adapting diabetes medication for low carbohydrate management of type 2 diabetes: a practical guide. Br J Gen Pract. (2019) 69:360–1. 10.3399/bjgp19X704525 31249097 PMC6592353

[12] Hallberg SJ Gershuni MVM Tamara L Hazbun TL Athinarayanan SJ. Reversing type 2 diabetes: a narrative review of the evidence. Nutrients. (2019) 11:766. 10.3390/nu11040766 31323831 PMC6683030

[13] Krauss R Blanche P Rawlings R Fernstrom H Williams P. Separate effects of reduced carbohydrate intake and weight loss on atherogenic dyslipidemia. Am J Clin Nutr. (2006) 83:1025–31. 10.1093/ajcn/83.5.1025 16685042

[14] Feinman RD Volek JS. Low carbohydrate diets improve atherogenic dyslipidemia even in the absence of weight loss. Nutr Metab. (2006) 3:24. 10.1186/1743-7075-3-24 16790045 PMC1488852

[15] Dyson P. Low carbohydrate diets and type 2 diabetes: what is the latest evidence? Diabetes Ther. (2015) 6:411–24. 10.1007/s13300-015-0136-9 26446553 PMC4674467

[16] Boden G Sargrad K Homko C Mozzoli M Stein TP. Effect of a low-carbohydrate diet on appetite, blood glucose levels and insulin resistance in obese patients with type 2 diabetics. Am Intern Med. (2005) 142:403–11. 10.7326/0003-4819-142-6-200503150-00006 15767618

[17] Johnstone AM Horgan GW Murison SD Bremner DM Lobley GE. Effects of a high-protein ketogenic diet on hunger, appetite, and weight loss in obese men feeding *ad libitum*. Am J Clin Nutr. (2008) 87:44–55. 10.1093/ajcn/87.1.44 18175736

[18] Gibson AA Seimon RV Lee CM Ayre J Franklin J Markovic TP. Do ketogenic diets really suppress appetite? A systematic review and meta-analysis. Obes Rev. (2015) 16:64–76. 10.1111/obr.12230 25402637

[19] Paoli A Bosco G Camporesi EM Mangar D. Ketosis, ketogenic diet and food intake control: a complex relationship. Front Psychol. (2015) 6:27. 10.3389/fpsyg.2015.00027 25698989 PMC4313585

[20] Foster GD Wyatt HR Hill JO McGuckin BG Brill C Mohammed BS. A randomized trial of a low-carbohydrate diet for obesity. N Engl J Med. (2003) 348:2082–90. 10.1056/NEJMoa022207 12761365

[21] Stern L Iqbal N Seshadri P Chicano KL Daily DA McGrory J. The effects of low-carbohydrate versus conventional weight loss diets in severely obese adults: one-year follow-up of a randomized trial. Ann Intern Med. (2004) 140:778–85. 10.7326/0003-4819-140-10-200405180-00007 15148064

[22] Westman EC Yancy WS Jr Mavropoulos JC Marquart M McDuffie JR. The effect of a low-carbohydrate, ketogenic diet versus a low-glycemic index diet on glycemic control in type 2 diabetes mellitus. Nutr Metab. (2008) 5:36. 10.1186/1743-7075-5-36 19099589 PMC2633336

[23] Foster GD Wyatt HR Hill JO Makris AP Rosenbaum DL Brill C. Weight and metabolic outcomes after 2 years on a low-carbohydrate versus low-fat diet: a randomized trial. Ann Intern Med. (2010) 153:147–57. 10.7326/0003-4819-153-3-201008030-00005 20679559 PMC2949959

[24] Goldstein T Kark JD Berry EM Adler B Ziv E Raz I. The effect of a low carbohydrate energy-unrestricted diet on weight loss in obese type 2 diabetes patients – a randomized controlled trial. e-SPEN. (2011) 6:e178–86. 10.1016/j.eclnm.2011.04.003

[25] Hall KD Guo J Courville AB Boring J Brychta R Chen KY. Effect of a plant-based, low-fat diet versus an animal-based, ketogenic diet on *ad libitum* energy intake. Nat Med. (2021) 27:344–53. 10.1038/s41591-020-01209-1 33479499

[26] Ludwig DS Dickinson SL Henschel B Ebbeling CB Allison DB. Do lower-carbohydrate diets increase total energy expenditure? An updated and reanalyzed meta-analysis of 29 controlled-feeding studies. J Nutr. (2020) 151:482–90. 10.1093/jn/nxaa350 33274750 PMC7948201

[27] Hall KD Ayuketah A Brychta R Cai H Cassimatis T Chen KY. Ultra-processed diets cause excess calorie intake and weight gain: an inpatient randomized controlled trial of *ad libitum* food intake. Cell Metab. (2019) 30:67–77. 10.1016/j.cmet.2019.05.008 31105044 PMC7946062

[28] Fuh MM-T Lee MM-S Jeng C-Y Ma F Chen Y-DI Reaven GM. Effect of low fat - high carbohydrate diets in hypertensive patients with non-insulin-dependent diabetes mellitus. Am J Hypertens. (1990) 3:527–32. 10.1093/ajh/3.7.527 2194509

[29] Brynes AE Edwards CM Ghatei MA Dornhorst A Morgan LM Bloom SR. A randomised four-intervention crossover study investigating the effect of carbohydrates on daytime profiles of insulin, glucose, non-esterified fatty acids and triacylglycerols in middle-aged men. Br J Nutr. (2003) 89:207–18. 10.1079/BJN2002769 12575905

[30] Ballard KD Quann EE Kupchak BR Volk BM Kawiecki DM Fernandez ML. Dietary carbohydrate restriction improves insulin sensitivity, blood pressure, microvascular function, and cellular adhesion markers in individuals taking statins. Nutr Res. (2013) 33:905–12. 10.1016/j.nutres.2013.07.022 24176230

[31] Gower BA Goss AM. A lower-carbohydrate, higher-fat diet reduces abdominal and intermuscular fat and increases insulin sensitivity in adults at risk of type 2 diabetes. J Nutr. (2015) 145:177–83S. 10.3945/jn.114.195065 25527677 PMC4264021

[32] Wiebe N Ye F Crumley ET Bello A Stenvinkel P Tonelli M. Temporal associations among body mass index, fasting insulin, and systemic inflammation: a systematic review and meta-analysis. JAMA Netw Open. (2021) 4:e211263. 10.1001/jamanetworkopen.2021.1263 33710289 PMC7955272

[33] Kolb H Stumvoll M Kramer W Kempf K Martin S. Insulin translates unfavourable lifestyle into obesity. BMC Med. (2018) 16:232. 10.1186/s12916-018-1225-1 30541568 PMC6292073

[34] Yalow RS Berson SA. Plasma insulin concentrations in nondiabetic and early diabetic subjects: determinations by a new sensitive immuno-assay technic. Diabetes. (1960) 9:254–60. 10.2337/diab.9.4.254 13846365

[35] Pories WJ Dohm GL. Diabetes: have we got it all wrong?: Hyperinsulinism as the culprit: surgery provides the evidence. Diabetes Care. (2012) 35:2438–42. 10.2337/dc12-0684 23173133 PMC3507594

[36] Hodish I. Insulin therapy, weight gain and prognosis. Diabetes Obesity Metab. (2018) 20:2085–92. 10.1111/dom.13367 29785843

[37] Korsmo-Haugen HK Brurberg KG Mann J Aas AM. Carbohydrate quantity in the dietary management of type 2 diabetes - a systematic review and meta-analysis. Diabetes Obesity Metab. (2018) 21:15–27. 10.1111/dom.13499 30098129

[38] Athinarayanan SJ Adams RN Hallberg SJ Mckenzie AL Bhanpuri NH Campbell WW. Long-term effects of a novel continuous remote care intervention including nutritional ketosis for the management of type 2 diabetes: a 2-year non-randomized clinical trial. Front Endocrinol. (2018) 10:348. 10.3389/fendo.2019.00348 PMC6561315 31231311

[39] Ludwig DS. The ketogenic diet: evidence for optimism but high-quality research needed. J Nutr. (2019) 150:1354–9. 10.1093/jn/nxz308 31825066 PMC7269727

[40] Walsh CO Ebbeling CB Swain JF Markowitz RL Feldman HA Ludwig DS. Effects of diet composition on postprandial energy availability during weight loss maintenance. PLoS ONE. (2013) 8:e58172. 10.1371/journal.pone.0058172 23483989 PMC3590159

[41] Shimy KJ Feldman HA Klein GL Bielak L Ebbeling CB Ludwig DS. Effects of dietary carbohydrate content on circulating metabolic fuel availability in the postprandial state. J Endocr Soc. (2020) 4:bvaa062. 10.1210/jendso/bvaa062 32666008 PMC7326475

[42] Ebbeling CB Feldman HA Klein GL Wong JMW Bielak L Steltz SK. Effects of a low carbohydrate diet on energy expenditure during weight loss maintenance: randomized trial. BMJ. (2018) 363:k4583. 10.1136/bmj.k4583 30429127 PMC6233655

[43] Gearhardt AN Davis C Kuschner R Brownell KD. The addiction potential of hyperpalatable foods. Curr Drug Abuse Rev. (2011) 4:140–5. 10.2174/1874473711104030140 21999688

[44] Johnson F Wardle J. Variety, palatability, and obesity. Adv Nutr Int Rev J. (2014) 5:851–9. 10.3945/an.114.007120 PMC4224225 25398751

[45] Aragon AA Schoenfeld BJ Wildman R Kleiner S VanDusseldorp T Taylor L. International society of sports nutrition position stand: diets and body composition. J Int Soc Sports Nutr. (2017) 14:16. 10.1186/s12970-017-0174-y 28630601 PMC5470183

[46] Gosby AK Conigrave AD Raubenheimer D Simpson SJ. Protein leverage and energy intake. Obes Rev. (2014) 15:183–91. 10.1111/obr.12131 24588967

[47] Simpson SJ Raubenheimer D. Obesity: the protein leverage hypothesis. Obes Rev. (2005) 6:133–42. 10.1111/j.1467-789X.2005.00178.x 15836464

[48] Hall KD. The potential role of protein leverage in the US obesity epidemic. Obesity. (2019) 27:1222–4. 10.1002/oby.22520 31095898 PMC7147114

[49] Brehm BJ Seeley RJ Daniels SR D'Alessio DA. A randomized trial comparing a very low carbohydrate diet and a calorie-restricted low fat diet on body weight and cardiovascular risk factors in healthy women. J Clin Endocrinol Metab. (2003) 88:1617–23. 10.1210/jc.2002-021480 12679447

[50] Yancy WS Jr Olsen MK Guyton JR Bakst RP Westman EC. A low-carbohydrate, ketogenic diet versus a low-fat diet to treat obesity and hyperlipidemia: a randomized, controlled trial. Ann Intern Med. (2004) 140:769–77. 10.7326/0003-4819-140-10-200405180-00006 15148063

[51] Gibson AA Sainsbury A. Strategies to improve adherence to dietary weight loss interventions in research and real-world settings. Behav Sci. (2017) 7:44. 10.3390/bs7030044 28696389 PMC5618052

[52] Manninen AH. Metabolic effects of the very-low-carbohydrate diets: misunderstood “Villains” of human metabolism. J Int Soc Sports Nutr. (2004) 1:7–11. 10.1186/1550-2783-1-2-7 18500949 PMC2129159

[53] Wu WC Wei JN Chen SC Fan KC Lin CH Yang CY. Progression of insulin resistance: a link between risk factors and the incidence of diabetes. Diabetes Res Clin Pract. (2020) 161:108050. 10.1016/j.diabres.2020.108050 32035116

[54] Sattar N Gill JMR. Type 2 diabetes as a disease of ectopic fat? BMC Med. (2014) 12:123. 10.1186/s12916-014-0123-4 25159817 PMC4143560

[55] Shanik MH Xu Y Skrha J Dankner R Zick Y Roth J. Insulin resistance and hyperinsulinemia: is hyperinsulinemia the cart or the horse? Diabetes Care. (2008) 31(Suppl. 2):S262–8. 10.2337/dc08-s264 18227495

[56] Cao W Liu H-Y Hong T Liu Z. Excess exposure to insulin may be the primary cause of insulin resistance. Am J Physiol Endocrinol Metab. (2010) 298:E372. 10.1152/ajpendo.00677.2009 20075432 PMC2822476

[57] Turner MC Martin NRW Player DJ Ferguson RA Wheeler P Green CJz. Characterising hyperinsulinaemia induced insulin resistance in human skeletal muscle cells. J Mol Endocrinol. (2020) 64:125–32. 10.1530/JME-19-0169 31990657

[58] Nolan CJ Prentki M. Insulin resistance and insulin hypersecretion in the metabolic syndrome and type 2 diabetes: time for a conceptual framework shift. Diabetes Vasc Dis Res. (2019) 13:118–27. 10.1177/1479164119827611 30770030

[59] Taylor R. Pathogenesis of type 2 diabetes: tracing the reverse route from cure to cause. Diabetologia. (2008) 51:1781–9. 10.1007/s00125-008-1116-7 18726585

[60] Stefan N Kantartzis K Haring HU. Causes and metabolic consequences of Fatty liver. Endocr Rev. (2008) 29:939–60. 10.1210/er.2008-0009 18723451

[61] Browning JD Baker JA Rogers T Davis J Satapati S Burgess SC. Short-term weight loss and hepatic triglyceride reduction: evidence of a metabolic advantage with dietary carbohydrate restriction. Am J Clin Nutr. (2011) 93:1048–52. 10.3945/ajcn.110.007674 21367948 PMC3076656

[62] Unwin D Tobin S. A patient request for some “deprescribing”. BMJ. (2015) 351:h4023. 10.1136/bmj.h4023 26239952

[63] Guess ND. Dietary interventions for the prevention of type 2 diabetes in high-risk groups: current state of evidence and future research needs. Nutrients. (2018) 10:1245. 10.3390/nu10091245 30200572 PMC6163866

[64] Gepner Y Shelef I Komy O Cohen N Schwarzfuchs D Bril N. The beneficial effects of Mediterranean diet over low-fat diet may be mediated by decreasing hepatic fat content. J Hepatol. (2019) 71:379–88. 10.1016/j.jhep.2019.04.013 31075323

[65] Luukkonen PK Dufour S Lyu K Zhang X-M Hakkarainen A Lehtimäki TE. Effect of a ketogenic diet on hepatic steatosis and hepatic mitochondrial metabolism in nonalcoholic fatty liver disease. Proc Nat Acad Sci. (2020) 117:7347–54. 10.1073/pnas.1922344117 32179679 PMC7132133

[66] Holmer M Lindqvist C Petersson S Moshtaghi-Svensson J Tillander V Brismar TB. Treatment of NAFLD with intermittent calorie restriction or low-carb high-fat diet – a randomized controlled trial. JHEP Rep. (2021) 3:100256. 10.1016/j.jhepr.2021.100256 33898960 PMC8059083

[67] Mardinoglu A Wu H Bjornson E Zhang C Hakkarainen A Räsänen SM. An integrated understanding of the rapid metabolic benefits of a carbohydrate-restricted diet on hepatic steatosis in humans. Cell Metab. (2018) 27:559–71. 10.1016/j.cmet.2018.01.005 29456073 PMC6706084

[68] Sanders FW Griffin JL. *De novo* lipogenesis in the liver in health and disease: more than just a shunting yard for glucose. Biol Rev. (2015) 91:452–68. 10.1111/brv.12178 25740151 PMC4832395

[69] Tappy L Le K. Metabolic effects of fructose and the worldwide increase in obesity. Physiol Rev. (2010) 90:23–46. 10.1152/physrev.00019.2009 20086073

[70] Al-Mrabeh A Zhyzhneuskaya SV Peter C Barnes AC Melhem S Jesuthasan A. Hepatic lipoprotein export and remission of human type 2 diabetes after weight loss. Cell Metab. (2019) 31:233–49. 10.1016/j.cmet.2019.11.018 31866441

[71] Taylor R Al-Mrabeh A Zhyzhneuskaya S Peters C Barnes AC Aribisala BS. Remission of human type 2 diabetes requires decrease in liver and pancreas fat content but is dependent upon capacity for β cell recovery. Cell Metab. (2018) 28:1–10. 10.1016/j.cmet.2018.08.010 30282047

[72] Eizirik DL Korbutt GS Hellerstrom C. Prolonged exposure of human pancreatic islets to high glucose concentrations *in vitro* impairs the beta-cell function. J Clin Invest. (1992) 90:1263–8. 10.1172/JCI115989 1401063 PMC443168

[73] Federici M Hribal M Perego L Ranalli M caradonna Z Perego C. High glucose causes apoptosis in cultured human pancreatic islets of langerhans: a potential role for regulation of specific bcl family genes toward an apoptotic cell death programe. Diabetes. (2001) 50:1290–301. 10.2337/diabetes.50.6.1290 11375329

[74] Robertson RP Harmon J Tran PO Tanaka Y Takahashi H. Glucose toxicity in beta-cells: type 2 diabetes, good radicals gone bad, and the glutathione connection. Diabetes. (2003) 52:581–7. 10.2337/diabetes.52.3.581 12606496

[75] Unwin DJ Tobin SD Murray SW Delon C Brady AJ. Substantial and sustained improvements in blood pressure, weight and lipid profiles from a carbohydrate restricted diet: an observational study of insulin resistant patients in primary care. Int J Environ Res Public Health. (2019) 16:2680. 10.3390/ijerph16152680 31357547 PMC6695889

[76] Tiwari S Riazi S Ecelbarger CA. Insulin's impact on renal sodium transport and blood pressure in health, obesity, and diabetes. Am J Physiol Renal Physiol. (2007) 293:F974–84. 10.1152/ajprenal.00149.2007 17686957

[77] Brands MW Manhiani MM. Sodium-retaining effect of insulin in diabetes. Am J Physiol Regul Integr Comp Physiol. (2012) 303:R1101–9. 10.1152/ajpregu.00390.2012 23034715 PMC3533616

[78] Harsha DW Bray GA. Weight loss and blood pressure control (Pro). Hypertension. (2008) 51:1420–5. 10.1161/HYPERTENSIONAHA.107.094011 18474829

[79] Cucuzzella M Hite A Patterson K Saslow L Heath R. A clinician's guide to inpatient low- carbohydrate diets for remission of type 2 diabetes: toward a standard of care protocol. Diabetes Management. (2019) 9:7–19. 10.37532/1758-1907.2019.9(1).7-19

[80] Bazzano L Cucuzzella M Westman E Yancy W. Low-Carbohydrate Nutrition Approaches in Patients with Obesity, Prediabetes and Type 2 Diabetes. (2019). Available online at: http://eguideline.guidelinecentral.com/i/1180534-low-carb-nutritional-approaches-guidelines-advisory/0 (accessed March 30, 2020).

[81] Volek JS Sharman MJ Forsythe CE. Modification of lipoproteins by very low-carbohydrate diets. J Nutr. (2005) 135:1339–42. 10.1093/jn/135.6.1339 15930434

[82] Bhanpuri NH Hallberg SJ Williams PT McKenzie AL Ballard KD Campbell WW. Cardiovascular disease risk factor responses to a type 2 diabetes care model including nutritional ketosis induced by sustained carbohydrate restriction at 1 year: an open label, non-randomized, controlled study. Cardiovasc Diabetol. (2018) 17:56. 10.1186/s12933-018-0698-8 29712560 PMC5928595

[83] van Wyk HJ Davis RE Davies JS. A critical review of low-carbohydrate diets in people with Type 2 diabetes. Diabetic Med. (2015) 33:148–57. 10.1111/dme.12964 26413954

[84] Iqbal N Vetter ML Moore RH Chittams JL Dalton-Bakes CV Dowd M. Effects of a low-intensity intervention that prescribed a low-carbohydrate vs. a low-fat diet in obese, diabetic participants. Obesity. (2010) 18:1733–8. 10.1038/oby.2009.460 20019677

[85] Gannon M Nuttall F Saeed A. An increase in dietary protein improves the blood glucose response in persons with type 2 diabetes. Am J Clin Nutr. (2003) 78:734–41. 10.1093/ajcn/78.4.734 14522731

[86] Gannon M Nuttall F. Effect of a high-protein, low-carbohydrate diet on blood glucose control in people with type 2 diabetes. Diabetes. (2004) 53:2375–82. 10.2337/diabetes.53.9.2375 15331548

[87] Nuttall FQ Gannon MC. Metabolic response of people with type 2 diabetes to a high protein diet. Nutr Metab. (2004) 1:6. 10.1186/1743-7075-1-6 15507157 PMC524031

[88] Sutton EF Beyl R Early KS Cefalu WT Ravussin E Peterson CM. Early time-restricted feeding improves insulin sensitivity, blood pressure, and oxidative stress even without weight loss in men with prediabetes. Cell Metab. (2018) 27:1–10. 10.1016/j.cmet.2018.04.010 29754952 PMC5990470

[89] Jakubowicz D Landau Z Tsameret S Wainstein J Raz I Ahren B. Reduction in glycated hemoglobin and daily insulin dose alongside circadian clock upregulation in patients with type 2 diabetes consuming a three-meal diet: a randomized clinical trial. Diabetes Care. (2019) 45:2171–80. 10.2337/dc19-1142 31548244

[90] Goldenberg JZ Day A Brinkworth GD Sato J Yamada S Jönsson T. Efficacy and safety of low and very low carbohydrate diets for type 2 diabetes remission: systematic review and meta-analysis of published and unpublished randomized trial data. BMJ. (2021) 372:m4743. 10.1136/bmj.m4743 33441384 PMC7804828

[91] Kodama S Saito K Tanaka S Maki M Yachi Y Sato M. Influence of fat and carbohydrate proportions on the metabolic profile in patients with type 2 diabetes: a meta-analysis. Diabetes Care. (2009) 32:959–65. 10.2337/dc08-1716 19407076 PMC2671123

[92] Ajala O English P Pinkney J. Systematic review and meta-analysis of different dietary approaches to the management of type 2 diabetes. Am J Clin Nutr. (2013) 97:505–16. 10.3945/ajcn.112.042457 23364002

[93] Naude CE Schoonees A Senekal M Young T Garner P Volmink J. Low carbohydrate versus isoenergetic balanced diets for reducing weight and cardiovascular risk: a systematic review and meta-analysis. PLoS ONE. (2014) 9:e100652. 10.1371/journal.pone.0100652 25007189 PMC4090010

[94] Fan Y Di H Chen G Mao X Liu C. Effects of low carbohydrate diets in individuals with type 2 diabetes: systematic review and meta-analysis. Int J Clin Exp Med. (2016) 9:11166–74. Available online at: www.ijcem.com/files/ijcem0023504.pdf

[95] Snorgaard O Poulsen GM Andersen HK Astrup A. Systematic review and meta-analysis of dietary carbohydrate restriction in patients with type 2 diabetes. BMJ Open Diabetes Res Care. (2017) 5:e000354. 10.1136/bmjdrc-2016-000354 28316796 PMC5337734

[96] Meng Y Bai H Wang S Li Z Wang Q Chen L. Efficacy of low carbohydrate diet for type 2 diabetes mellitus management: a systematic review and meta-analysis of randomized controlled trials. Diabetes Res Clin Pract. (2017) 131:124–31. 10.1016/j.diabres.2017.07.006 28750216

[97] Sainsbury E Kizirian NV Partridge SR Gill T Colagiuri S Gibson AA. Effect of dietary carbohydrate restriction on glycemic control in adults with diabetes: a systematic review and meta-analysis. Diabetes Res Clin Pract. (2018) 139:239–52. 10.1016/j.diabres.2018.02.026 29522789

[98] van Zuuren EJ Fedorowicz Z Kuijpers T Pijl H. Effects of low-carbohydrate- compared with low-fat-diet interventions on metabolic control in people with type 2 diabetes: a systematic review including GRADE assessments. Am J Clin Nutr. (2018) 108:1–32. 10.1093/ajcn/nqy096 30007275

[99] Huntriss R Campbell M Bedwell C. The interpretation and effect of a low-carbohydrate diet in the management of type 2 diabetes: a systematic review and meta-analysis of randomised controlled trials. Eur J Clin Nutr. (2018) 72:311–25. 10.1038/s41430-017-0019-4 29269890

[100] McArdle PD Greenfield SM Rilstone SK Narendran P Haque MS Gill PS. Carbohydrate restriction for glycaemic control in Type 2 diabetes: a systematic review and meta-analysis. Diabetic Med. (2019) 36:335–48. 10.1111/dme.13862 30426553

[101] Nagi D Hambling C Taylor R. Remission of type 2 diabetes: a position statement from the Association of British Clinical Diabetologists (ABCD) and the Primary Care Diabetes Society (PCDS). Br J Diabetes. (2019) 19:73–6. 10.15277/bjd.2019.221

[102] Gumbiner B Low CC Reaven PD. Effects of a monounsaturated fatty acid-enriched hypocaloric diet on cardiovascular risk factors in obese patients with type 2 diabetes. Diabetes Care. (1998) 21:9–15. 10.2337/diacare.21.1.9 9538963

[103] Nielsen JV Jonsson E Nilsson AK. Lasting improvement of hyperglycaemia and bodyweight: low-carbohydrate diet in type 2 diabetes – a brief report. Ups J Med Sci. (2005) 110:179–83. 10.3109/2000-1967-182 16075898

[104] Dyson PA Beatty S Matthews DR. A low-carbohydrate diet is more effective in reducing body weight than healthy eating in both diabetic and non-diabetic subjects. Diabetic Med. Oxford (2007) 24:1430–5. 10.1111/j.1464-5491.2007.02290.x 17971178

[105] Shai I Schwarzfuchs D Henkin Y Shahar DR Witkow S Greenberg I. Weight loss with a low-carbohydrate, Mediterranean, or low-fat diet. N Engl J Med. (2008) 359:229–41. 10.1056/NEJMoa0708681 18635428

[106] Dyson PA. Dietary Advice for People With Diabetes: The Role of Carbohydrate in Dietary Treatment and an Assessment of Video Education. Oxford: Oxford Brookes University (2010).

[107] Yancy WS Jr Westman EC McDuffie JR. A randomized trial of a low-carbohydrate diet vs orlistat plus a low-fat diet for weight loss. Arch Internal Med. (2010) 170:136–45. 10.1001/archinternmed.2009.492 20101008

[108] Mayer SB Jeffreys AS Olsen MK McDuffie JR Feinglos MN Yancy WS. Two diets with different hemoglobin A(1c) and antiglycemic medication effects despite similar weight loss in type 2 diabetes. Diabetes Obes Metab. (2014) 16:90–3. 10.1111/dom.12191 23911112 PMC3867584

[109] Saslow LR Kim S Daubenmier JJ Moskowitz JT Phinney SD Goldman V. A randomized pilot trial of a moderate carbohydrate diet compared to a very low carbohydrate diet in overweight or obese individuals with type 2 diabetes mellitus or prediabetes. PLoS ONE. (2014) 9:e91027. 10.1371/journal.pone.0091027 24717684 PMC3981696

[110] Yamada Y Uchida J Izumi H Tsukamoto Y Inoue G Watanabe Y. A non-calorie-restricted low-carbohydrate diet is effective as an alternative therapy for patients with type 2 diabetes. Intern Med. (2014) 53:13–9. 10.2169/internalmedicine.53.0861 24390522

[111] Saslow LR Mason AE Kim S Goldman V Ploutz-Snyder R Bayandorian H. An online intervention comparing a very low-carbohydrate ketogenic diet and lifestyle recommendations versus a plate method diet in overweight individuals with type 2 diabetes: a randomized controlled trial. J Med Internet Res. (2017) 19:e36. 10.2196/jmir.5806 28193599 PMC5329646

[112] Nishimori E Ogata S Naka M. Comparison of effects of low-carbohydrate diet and calorie-restricted diet on nonalcoholic fatty liver disease in japanese patients with type 2 diabetes. Diabetes. (2018) 67(Suppl. 1):761. 10.2337/db18-761-P

[113] Asle Mohammadi Zadeh M Kargarfard M Marandi SM Habibi A. Diets along with interval training regimes improves inflammatory & anti-inflammatory condition in obesity with type 2 diabetes subjects. J Diabetes Metab Disord. (2018) 17:253–67. 10.1007/s40200-018-0368-0 30918861 PMC6405404

[114] Morris E Aveyard P Dyson P Noreik M Bailey C Fox R. A food-based low-energy, low-carbohydrate diet for people with type 2 diabetes in primary care: a randomised controlled feasibility trial. Diabetes Obes Metab. (2019) 22:512–20. 10.1111/dom.13915 31709697 PMC7079070

[115] Breukelman GJ Basson AK Djarova TG Du Preez CJ Shaw I Shaw BS. Combination low carbohydrate, high fat diet and physical activity intervention on lipoprotein-lipids in type 2 diabetics. Asian J Sports Med. (2019) 10:e86905. 10.5812/asjsm.86905

[116] Perna S Alalwan TA Gozzer C Infantino V Peroni G Gasparri C. Effectiveness of a hypocaloric and low-carbohydrate diet on visceral adipose tissue and glycemic control in overweight and obese patients with type 2 diabetes. Bahrain Med Bull. (2019) 41:159–64. Available online at: https://www.bahrainmedicalbulletin.com/SEPT_2019/SEPT2019_EFFECTIVENESS.pdf

[117] Goday A Bellido D Sajoux I Crujeiras AB Burguera B Garcia-Luna PP. Short-term safety, tolerability and efficacy of a very low-calorie-ketogenic diet interventional weight loss program versus hypocaloric diet in patients with type 2 diabetes mellitus. Nutr Diabetes. (2016) 6:e230. 10.1038/nutd.2016.36 27643725 PMC5048014

[118] Vlachos D Ganotopoulou A Stathi C Koutsovasilis A Diakoumopoulou E Doulgerakis D. A low-carbohydrate protein sparing modified fast diet compared with a low glycaemic index reduced calorie diet in obese type 2 diabetic patients. Diabetologia. (2011) 54:S355. 10.1007/s00125-011-2276-4

[119] Lee P Paisey RB Waterson M Daly ME Gale TJ Williams K. Reduction in High Sensitivity C-reactive Protein levels in type 2 diabetes after low carbohydrate but not energy deficit diet. Diabetic Med. (2013) 30:47. 10.1111/dme.12091_1

[120] Davis NJ Tomuta N Schechter C Isasi CR Segal-Isaacson CJ Stein D. Comparative study of the effects of a 1-year dietary intervention of a low-carbohydrate diet versus a low-fat diet on weight and glycemic control in type 2 diabetes. Diabetes Care. (2009) 32:1147–52. 10.2337/dc08-2108 19366978 PMC2699720

[121] Sato J Kanazawa A Makita S Hatae C Komiya K Shimizu T. A randomized controlled trial of 130 g/day low-carbohydrate diet in type 2 diabetes with poor glycemic control. Clin Nutr. (2017) 36:992–1000. 10.1016/j.clnu.2016.07.003 27472929

[122] Samaha FF Iqbal N Seshadri P Chicano KL Daily DA McGrory J. A low-carbohydrate as compared with a low-fat diet in severe obesity. N Engl J Med. (2003) 348:2074–81. 10.1056/NEJMoa022637 12761364

[123] Daly ME Paisey R Paisey R Millward BA Eccles C Williams K. Short-term effects of severe dietary carbohydrate-restriction advice in Type 2 diabetes—a randomized controlled trial. Diabetic Med. (2006) 23:15–20. 10.1111/j.1464-5491.2005.01760.x 16409560

[124] Guldbrand H Dizdar B Bunjaku B Lindström T Bachrach-Lindström M Fredrikson M. In type 2 diabetes, randomisation to advice to follow a low-carbohydrate diet transiently improves glycaemic control compared with advice to follow a low-fat diet producing a similar weight loss. Diabetologia. (2012) 55:2118–27. 10.1007/s00125-012-2567-4 22562179 PMC3390696

[125] Jonasson L Guldbrand H Lundberg AK Nystrom FH. Advice to follow a low-carbohydrate diet has a favourable impact on low-grade inflammation in type 2 diabetes compared with advice to follow a low-fat diet. Ann Med. (2014) 46:182–7. 10.3109/07853890.2014.894286 24779961 PMC4025600

[126] Tay J Natalie DL-M Thompson CH Noakes M Buckley JD Wittert GA. A very low carbohydrate, low saturated fat diet for type 2 diabetes management: a randomized trial. Diabetes Care. (2014) 37:2909–18. 10.2337/dc14-0845 25071075

[127] Tay J Luscombe-Marsh ND Thompson CH Noakes M Buckley JD Wittert GA. Comparison of low- and high-carbohydrate diets for type 2 diabetes management: a randomized trial. Am J Clin Nutr. (2015) 102:780–90. 10.3945/ajcn.115.112581 26224300

[128] Tay J Thompson CH Luscombe-Marsh ND Wycherley TP Noakes M Buckley JD. Effects of an energy-restricted low-carbohydrate, high unsaturated fat/low saturated fat diet versus a high carbohydrate, low fat diet in type 2 diabetes: a 2 year randomized clinical trial. Diabetes Obes Metab. (2018) 20:858–71. 10.1111/dom.13164 29178536

[129] Lean MEJ Leslie WS Barnes AC Brosnahan N Thom G McCombie L. Durability of a primary care-led weight-management intervention for remission of type 2 diabetes: 2-year results of the DiRECT open-label, cluster-randomised trial. Lancet Diabetes Endocrinol. (2019) 7:344–55. 10.1016/S2213-8587(19)30068-3 30852132

[130] Hallberg SJ McKenzie AL Williams PT Bhanpuri NH Peters AL Campbell WW. Effectiveness and safety of a novel care model for the management of type 2 diabetes at 1 year: an open-label, non-randomized, controlled study. Diabetes Ther. (2018) 9:583–612. 10.1007/s13300-018-0373-9 29417495 PMC6104272

[131] Athinarayanan SJ Hallberg SJ McKenzie AL Lechner K King S McCarter JP. Impact of a 2-year trial of nutritional ketosis on indices of cardiovascular disease risk in patients with type 2 diabetes. Cardiovasc Diabetol. (2020) 19:208. 10.1186/s12933-020-01178-2 33292205 PMC7724865

[132] Saslow LR Summers C Aikens JE Unwin DJ. Outcomes of a digitally delivered low-carbohydrate type 2 diabetes self-management program: 1-year results of a single-arm longitudinal study. JMIR Diabetes. (2018) 3:e12. 10.2196/diabetes.9333 30291081 PMC6238840

[133] Unwin D Unwin J. Low carbohydrate diet to achieve weight loss and improve HbA1c in type 2 diabetes and pre-diabetes: experience from one general practice. Pract Diabetes. (2014) 31:76–9. 10.1002/pdi.1835

[134] Unwin DJ Cuthbertson DJ Feinman R Sprung VS. A pilot study to explore the role of a low carbohydrate intervention to improve GGT levels and HbA1c. Diabesity Pract. (2015) 4:102–8. Available online at: https://diabetesonthenet.com/diabetes-practice/a-pilot-study-to-explore-the-role-of-a-low-carbohydrate-intervention-to-improve-ggt-levels-and-hba1c/

[135] Unwin D Khalid AA Unwin J Crocombe D Delon C Martyn K. Insights from a general practice service evaluation supporting a lower carbohydrate diet in patients with type 2 diabetes mellitus and prediabetes: a secondary analysis of routine clinic data including HbA1c, weight and prescribing over 6 years. BMJ Nutr Prev Health. (2020) 3:285–94. 10.1136/bmjnph-2020-000072 33521540 PMC7841829

[136] Unwin D Haslam D Livesey G. It is the glycaemic response to, not the carbohydrate content of food that matters in diabetes and obesity: the glycaemic index revisited. J Insulin Resist. (2016) 1:a8. 10.4102/jir.v1i1.8

[137] Ahmed SR Bellamkonda S Zilbermint M Wang J Kalyani RR. Effects of the low carbohydrate, high fat diet on glycemic control and body weight in patients with type 2 diabetes: experience from a community-based cohort. BMJ Open Diabetes Res Care. (2020) 8:e000980. 10.1136/bmjdrc-2019-000980 32193200 PMC7103851

[138] Dyson PA Kelly T Deakin T Duncan A Frost G Harrison Z. Diabetes UK evidence-based nutrition guidelines for the prevention and management of diabetes. Diabet Med. (2011) 28:1282–8. 10.1111/j.1464-5491.2011.03371.x 21699560

[139] Dyson PA Twenefour D Breen C Duncan A Elvin E Goff L. Diabetes UK evidence-based nutrition guidelines for the prevention and management of diabetes. Diabet Med. (2018) 35:541–7. 10.1111/dme.13603 29443421

[140] Diabetes UK. Position Statement: Low-Carb Diets for People With Diabetes (2017).

[141] British Dietetic Association. Policy Statement - Low Carbohydrate Diets for the Management of Type 2 Diabetes in Adults (2018).

[142] Scottish Intercollegiate Guidelines Network. Management of Diabetes: A National Clinical Guideline (2010).

[143] Davies MJ D'Alessio DA Fradkin J Kernan WN Mathieu C Mingrone G. Management of Hyperglycemia in Type 2 Diabetes, 2018. A Consensus Report by the American Diabetes Association (ADA) and the European Association for the Study of Diabetes (EASD). Diabetes Care. (2018) 41:2669–701. 10.2337/dci18-0033 30291106 PMC6245208

[144] American Diabetes Association. 5. Facilitating behavior change and well-being to improve health outcomes: standards of medical care in diabetes-2020. Diabetes Care. (2020) 43(Suppl. 1):S48–65. 10.2337/dc20-S005 31862748

[145] National Institute for Health and Care Excellence. Type 2 Diabetes in Adults: Management. (2015). Available online at: https://www.nice.org.uk/guidance/ng28 (accessed January 27, 2020).

[146] Public Health England. The Eatwell Guide Helping You Eat a Healthy, Balanced Diet. (2016).

[147] National Institute for Health and Care Excellence. Surveillance of Type 2 Diabetes in Adults: Management 2015 - Appendix B2: Stakeholder Consultation, (2019). Available online at: https://www.nice.org.uk/guidance/ng28/evidence/appendix-b2-stakeholder-consultation-comments-table-ng28-pdf-6837997937 (accessed March 24, 2020).

[148] Shikany JM Margolis KL Pettinger M Jackson RD Limacher MC Liu S. Effects of a low-fat dietary intervention on glucose, insulin, and insulin resistance in the Women's Health Initiative (WHI) Dietary Modification trial. Am J Clin Nutr. (2011) 94:75–85. 10.3945/ajcn.110.010843 21562091 PMC3127523

[149] The Look AHEAD Research Group. Cardiovascular effects of intensive lifestyle intervention in type 2 diabetes. N Eng J Med. (2013) 369:145–54. 10.1056/NEJMoa1212914 PMC3791615 23796131

[150] Churuangsuk C Lean MEJ Combet E. Lower carbohydrate and higher fat intakes are associated with higher hemoglobin A1c: findings from the UK National Diet and Nutrition Survey 2008-2016. Eur J Nutr. (2019) 59:2771–82. 10.1007/s00394-019-02122-1 31686204 PMC7413867

[151] Seidelmann SB Claggett B Cheng S Henglin M Shah A Steffen LM. Dietary carbohydrate intake and mortality: a prospective cohort study and meta-analysis. Lancet Public Health. (2018) 3:E419–28. 10.1016/S2468-2667(18)30135-X 30122560 PMC6339822

[152] Mazidi M Katsiki N Mikhailidis DP Sattar N Banach M. Lower carbohydrate diets and all-cause and cause-specific mortality: a population-based cohort study and pooling of prospective studies. Eur Heart J. (2019) 40:2870–9. 10.1093/eurheartj/ehz174 31004146

[153] Rayner J D'Arcy E Ross LJ Hodge A Schoenaker DAJM. Carbohydrate restriction in midlife is associated with higher risk of type 2 diabetes among Australian women: a cohort study. Nutr Metab Cardiovasc Dis. (2020) 30:400–9. 10.1016/j.numecd.2019.11.001 31822429

[154] Shan Z Guo Y Hu FB Liu L Qi Q. Association of low-carbohydrate and low-fat diets with mortality among US Adults. JAMA Internal Med. (2020) 180:513–23. 10.1001/jamainternmed.2019.6980 31961383 PMC6990856

[155] Boniface S Kneale J Shelton N. Drinking pattern is more strongly associated with under-reporting of alcohol consumption than socio-demographic factors: evidence from a mixed-methods study. BMC Public Health. (2014) 14:1297. 10.1186/1471-2458-14-1297 25519144 PMC4320509

[156] Liangpunsakul S. Relationship between alcohol intake and dietary pattern: findings from NHANES III. World J Gastroenterol. (2010) 16:4055–60. 10.3748/wjg.v16.i32.4055 20731019 PMC2928459

[157] Kelly T Unwin D Finucane F. Low-carbohydrate diets in the management of obesity and type 2 diabetes: a review from clinicians using the approach in practice. Int J Environ Res Public Health. (2020) 17:2557. 10.3390/ijerph17072557 32276484 PMC7177487

[158] Dugani SB Moorthy MV Li C Demler OV Alsheikh-Ali AA Ridker PM. Association of lipid, inflammatory, and metabolic biomarkers with age at onset for incident coronary heart disease in women. JAMA Cardiol. (2021) 6:437–47. 10.1001/jamacardio.2020.7073 33471027 PMC7818181

[159] Johannesen CDL Mortensen MB Langsted A Nordestgaard BG. Apolipoprotein B and non-HDL cholesterol better reflect residual risk than LDL cholesterol in statin-treated patients. J Am Coll Cardiol. (2021) 77:1439–50. 10.1016/j.jacc.2021.01.027 33736827

[160] Zinöcker MK Svendsen K Dankel SN. The homeoviscous adaptation to dietary lipids (HADL) model explains controversies over saturated fat, cholesterol, and cardiovascular disease risk. Am J Clin Nutr. (2021) 113:277–89. 10.1093/ajcn/nqaa322 33471045

[161] Skogsberg J Dicker A Ryden M Astrom G Nilsson R Bhuiyan H. ApoB100-LDL acts as a metabolic signal from liver to peripheral fat causing inhibition of lipolysis in adipocytes. PLoS ONE. (2008) 3:e3771. 10.1371/journal.pone.0003771 19020660 PMC2582480

[162] Huntriss R Boocock R McArdle P. Dietary carbohydrate restriction as a management strategy for adults with type 2 diabetes: exploring the opinions of dietitians. J Diabetes Nurs. (2019) 23:JDN104. Available online at: https://diabetesonthenet.com/wp-content/uploads/pdf/dotn5ea6d21c2dab91c6e4476de47c690fa0.pdf

[163] Wong K Raffray M Roy-Fleming A Blunden S Brazeau A-S. Ketogenic diet as a normal way of eating in adults with type 1 and type 2 diabetes: a qualitative study. Can J Diabetes. (2021) 45:137–43.e1. 10.1016/j.jcjd.2020.06.016 33039330

[164] Zinn C Rush A Johnson R. Assessing the nutrient intake of a low-carbohydrate, high-fat (LCHF) diet: a hypothetical case study design. BMJ Open. (2018) 8:e018846. 10.1136/bmjopen-2017-018846 29439004 PMC5829852

[165] Zinn C North S Donovan K Muir C Henderson G. Low-carbohydrate, healthy-fat eating: a cost comparison with national dietary guidelines. Nutr Diet. (2019). 10.1111/1747-0080.12534 31020780 PMC7187181

[166] Esposito K Maiorino MI Petrizzo M Bellastella G Giugliano D. The effects of a mediterranean diet on the need for diabetes drugs and remission of newly diagnosed type 2 diabetes: follow-up of a randomized trial. Diabetes Care. (2014) 37:1824–30. 10.2337/dc13-2899 24722497

[167] Gregg EW Chen H Wagenknecht LE Clark JM Delahanty LM Bantle J. Association of an intensive lifestyle intervention with remission of type 2 diabetes. JAMA. (2012) 308:2489–96. 10.1001/jama.2012.67929 23288372 PMC4771522

[168] Ried-Larsen M Johansen MY MacDonald CS Hansen KB Christensen R Wedell-Neergaard AS. Type 2 diabetes remission 1 year after an intensive lifestyle intervention: a secondary analysis of a randomized clinical trial. Diabetes Obes Metab. (2019) 21:2257–66. 10.1111/dom.13802 31168922 PMC6772176

[169] Dave R Davis R Davies JS. The impact of multiple lifestyle interventions on remission of type 2 diabetes mellitus within a clinical setting. Obes Med. (2019) 13:59–64. 10.1016/j.obmed.2019.01.005

[170] Lean MEJ Leslie WS Barnes AC Brosnahan N Thom G McCombie L. Primary care-led weight management for remission of type 2 diabetes (DiRECT): an open-label, cluster-randomised trial. Lancet. (2017) 391:541–51. 10.1016/S0140-6736(17)33102-1 29221645

[171] Steven S Taylor R. Restoring normoglycaemia by use of a very low calorie diet in long- and short-duration Type 2 diabetes. Diabetic Med. (2015) 32:1149–55. 10.1111/dme.12722 25683066

