# Supplementary material for: Low Carbohydrate Dietary Approaches for People With Type 2 Diabetes—A Narrative Review
Source: Front Nutr. 2021 Jul 15;8:687658. doi: 10.3389/fnut.2021.687658 (PMC8319397; doi:10.3389/fnut.2021.687658)
Supplement: Supplementary file 1 [file Table_1.DOCX]

**Supplementary Table 1. Systematic reviews with meta-analyses comparing low carbohydrate dietary approaches* with higher carbohydrate dietary approaches for the management of Type 2 diabetes**

| **Article reference**** | **Inclusion criteria** | **Number of studies/participants** | **Assessment of included studies’ quality** | **Key outcomes** | **Limitations** |
| --- | --- | --- | --- | --- | --- |
| Kodama et al 2009(1) | Studies published in English up to 2007  RCTs comparing low carbohydrate high fat and high carbohydrate low fat diets with no difference in energy and protein, in people with Type 2 diabetes | 19 publications, which included 22 trials (some had multiple trials reported within a single publication) included in meta-analyses (n = 306) | 10 described drop-out rates and 9 didn't; drop-out ranged from 0 to 25%  None of the articles described methods of randomisation, so all had low quality scores  Possible publication bias for HDL-c (Egger’s test, p = 0.08; recommended level of significance, p = 0.10) | There were no significant differences in HbA1c change between diets  Two-hour glucose and insulin values were higher in the low fat group than in the low carbohydrate group by 10.3% (95%CI 6.7 to 13.9%; p < 0.001) and 12.8% (95%CI 5.2 to 20.4%; p < 0.001), respectively  The low fat diet also resulted in significant increases in TG (13.4%, 95%CI 7.1 to 19.8%; p < 0.001) and a significant reduction in HDL-c (5.6%, 95%CI 2.9 to 8.4%; p < 0.001) compared with that associated with the low carbohydrate diet  There were no significant differences in TC or LDL-c changes between diets | Limited details of methods presented and the definitions of diets is vague  Carbohydrate intake in the lower carb groups ranged from 30 to 50% total energy, so none were low carbohydrate diets by definitions applied within current review  Requirement for protein and energy intake to be comparable between groups limits scope for natural effects of diets to be assessed  Very short-term, studies ranged from 10 days to 6 weeks  No heterogeneity data presented  All studies were deemed to be low quality  Of the 17 studies with a crossover design, 9 (covering 10 trials) did not include a washout period, which could lead to an underestimation of differences due to a carryover effect |
| Ajala et al 2013(2) | Studies up to July 2011  No explicit mention of whether there were any language restrictions  RCTs in adults with an intervention that lasted ≥ 6 months, that compared any of the following diets with any control diet in people with Type 2 diabetes:   - Low carbohydrate - Low glycaemic index - High fibre - High protein - Vegetarian - Vegan - Mediterranean | 20 studies included in qualitative analyses (n = 3,460). 9 studies compared a low carbohydrate diet to control, 8 of which were included in the relevant meta-analyses | Quality assessment not reported separately for low carbohydrate studies.  For all studies:  3/16 studies didn't report method of randomisation  10/16 didn't report method of allocation concealment  6/16 were analysed on intention to treat basis.  None of the trials reported any significant differences in participant characteristics between groups at baseline | Low carbohydrate diets significantly decreased HbA1c compared with other diets (WMD: -0.12%, 95% CI -0.24 to 0.00, p = 0.04, I^2^ = 75%)  There was no difference in weight loss for low carbohydrate diets compared with control diets (P = 0.21; though the number of studies or participants was not reported, no forest plot was included, and I^2^ was not reported)  Low carbohydrate diets appeared to be beneficial for increasing HDL-c (WMD: 0.08 mmol/L, 95%CI 0.05 to 0.11, p < 0.00001) with no significant difference in LDL-c (P = 0.57) or TG (P = 0.47); though, again, the number of studies or participants was not reported, no forest plot was included, and I^2^ was not reported | There is a lack of detail, making it difficult to appraise the results, and "low carb" is not defined in inclusion criteria (discussion says low carbohydrate diets restrict carbohydrate intake to 20-60 g/d but this is not how it was defined in included studies, which included target carbohydrate intakes of up to 45%)  Both Samaha et al 2003(3) and Stern et al 2004(4) were included in meta-analyses despite reporting results from the same study  High heterogeneity for low carbohydrate glycaemic control meta-analysis, and I^2^ was not reported for weight and lipid analyses  Study durations ranging from 6 months to 4 years, but no attempt to consider effect of this  Includes Iqbal et al 2010(5), where there was no difference in macronutrient intake between groups (and carbohydrate intake was actually higher in the control arm at multiple time points) |
| Naude et al 2014(6) | Studies published in English up to 19th March 2014  RCTs with more than 10 participants in each group, adults only  Review not specific to diabetes but analysis limited to people with Type 2 diabetes presented  Compared low carbohydrate diets (defined as ≤45% total energy from carbs, with high fat or high protein) with control  Prescribed energy content had to be “the same or similar” in intervention and control groups | 5 studies in people with Type 2 diabetes were included in meta-analyses (n=660; 61 in 1 study assessing low carbohydrate, high fat and 599 in 4 studies assessing low carbohydrate, high protein) | For randomisation 4 studies were low risk and 1 was unclear risk  For allocation concealment 2 were low risk and 3 unclear risk  For performance bias all had unclear risk  For detection bias 1 was low risk and 4 were unclear risk  For attrition bias 3 were high risk, 1 was low risk and 1 was unclear risk  For reporting bias 2 were high risk and 3 were low risk  For "other" sources of bias 3 were high risk, 1 was low risk and 1 was unclear risk | There were no significant differences in HbA1c change between diets at 3-6 months (0.19%, 95%CI 0.00 to 0.39; 5 trials) or 1-2 years (0.01%, 95%CI -0.28 to 0.30, 4 trials)  No differences between diets for fasting blood glucose were noted, but few studies assessed this (1 at 3-6 months, 2 at 15 months)  There were no significant differences in body weight change between diets at 3-6 months (0.82 kg, 95%CI -1.25 to 2.90; 5 trials) or 1-2 years (0.91 kg, 95%CI -2.08 to 3.89; 4 trials)  There were no differences in changes in SBP or DBP at 3-6 months (SBP: 95%CI -3.14 to 4.36 mmHg; 4 trials. DBP: 95%CI -1.77 to 3.30 mmHg; 4 trials) or 1-2 years (SBP: 95%CI -3.10 to 3.72 mmHg; 4 trials. DBP: 95%CI -1.95 to 2.13 mmHg, 4 trials)  At 3–6 months, changes from baseline in blood lipids (TC, LDL-c, HDL-c and TG) were inconsistent and the changes in the meta-analyses were small, suggesting little or no difference in effect between the two diets  In 3 of the 4 studies with relevant data in people with Type 2 diabetes, adherence (based on an arbitrary score derived by the study authors) was better for the low carbohydrate diet than the control diet | Only isoenergetic studies were included, which precludes the assessment of the real world effects of the diets  High threshold for classifying low carbohydrate, in relation to the definitions used within the current review, and no sensitivity analyses  Only 5 studies included in Type 2 diabetes specific analyses (< 600 participants for all meta-analyses at 3-6 months, < 500 for all at 1-2 years)  Quality of evidence (assessed using GRADE) was low to moderate for all studies  Subject of a published critique (Harcombe and Noakes 2016(7)), which identified a number of flaws – most of which were deemed to penalise the low carbohydrate interventions |
| Fan et al 2016 (8) | Studies up to 30^th^ May 2014 (no language restriction)  RCTs in adults  All participants with Type 2 diabetes  One group receiving a low carbohydrate diet, defined as max 130g/day, compared to any other diet | 25 studies included in qualitative analysis, 10 studies included in meta-analyses (n = 1,080) | All studies except one were deemed to be of good quality (classed as having a Jadad score of 3 or higher) | There was a significant decrease in HbA1c in subjects who consumed low carbohydrate diets compared with other diets (WMD: -0.33%, 95%CI -0.51 to -0.15; p < 0.001, I^2^ = 88.4%; 10 studies)  Low carbohydrate diets were more effective for achieving weight loss than control diets (WMD: -2.4 Kg, 95%CI -3.7 to -1.1, p < 0.001; 10 studies). I^2^ was not reported for the WMD reported in the text, though was high for other analyses of weight change presented in forest plots (87.1 to 94.4%, except for data at 12 months where I^2^ reported as 0.0%).  Low-carbohydrate diets appeared to be beneficial in increasing HDL-c (WMD: 0.094 mmol/L, 95% CI 0.043 to 0.144, p < 0.001) and decreasing TG (WMD: -0.28 mmol/L, 95% CI 0.393 to -0.167, p < 0.001) with no significant reduction in TC (WMD: 0.051 mmol/L; 95% CI -0.144, 0.246; p = 0.61) or LDL-c (WMD: -0.027 mmol/L, 95% CI -0.108 to 0.053, p = 0.508). It was unclear how many studies each of these analyses was based on, and I^2^ was not reported for these outcomes | High heterogeneity where reported (missing for some outcomes)  Unclear how many studies were included in many of the meta-analyses  Includes Iqbal et al 2010(5), where there was no difference in macronutrient intake between groups (and carbohydrate intake was actually higher in the control arm at multiple time points) |
| Snorgaard et al 2017(9) | RCTs published in English or Scandinavian languages between January 2004 and October 2014  Population was subjects with Type 2 diabetes (doesn't explicitly state adults)  Comparing carbohydrate restriction (defined as <45% total energy) to control diets (with carbohydrate intake of 45-60%)  Primary outcomes were HbA1c or BMI after 1 year or more, secondary outcomes same markers but less than 1 year - plus LDL-c and dropout rates | 10 studies included in meta-analyses (n = 1,376) | Possible attrition bias, but no other potential sources of bias identified. Concluded that the overall risk of bias was low to moderate | In the low carbohydrate diets mean HbA1c was 0.34% lower in studies shorter than 1 year (95%CI -0.63 to -0.06; 8 studies, n = 809). There was no difference in studies that were longer than 1 year (7 studies, n = 839)  Meta-regression demonstrated a reduction in HbA1c as carbohydrate intake decreased (r = -0.85, p < 0.01; 8 studies), though shorter- and longer-term data were combined. The two studies with the lowest daily carbohydrate intake showed the largest reductions in HbA1c  Mean BMI in studies shorter than 1 year was 1.02 kg/m^2^ lower after the low carbohydrate diets (95%CI -2.58 to +0.54; 4 studies, n = 185) and was 0.43 kg/m^2^ lower in studies that were longer than 1 year (95%CI -1.38 to +0.53; 2 studies, n = 159). Statistical significance was not reported.  Reports on glucose lowering medication were available in seven studies. Medication reduction was significantly greater at 3 months and 6 months, and was “numerically lower” at 12 months  There were no differences between groups for the number of reported adverse events, cardiovascular events, or mortality | Average reported carbohydrate intake was 30% at 1 year in the five studies that reported this (range = 27% to 45%, so were ALL above threshold used to define low carbohydrate diets in the current review) and was even higher at 24 months (mean 31%, 45% and 48% respectively in the 3 trials that reported this)  Number of participants within each meta-analysis was small  High heterogeneity for HbA1c at less than a year (I^2^ = 74%). No heterogeneity data presented for outcomes other than HbA1c  Includes Iqbal et al 2010(5), where there was no difference in macronutrient intake between groups (and carbohydrate intake was actually higher in the control arm at multiple time points)  Included Saslow et al 2014(10) even though not all participants had Type 2 diabetes (it is also of note that carbohydrate intake was significantly reduced in the control group of this study, from a mean of 224g/day to 160g/day)  In the discussion of Korsmo-Haugen et al 2018(11) (see below) it is asserted that the relationship observed in the regression analysis of this review was "totally dependent on the findings of two trials with a duration of 3 months that were not included in our analyses because they involved participants with prediabetes or an additional physical activity intervention." |
| Korsmo-Haugen et al 2018(11) | Studies published in English, Danish, Norwegian and Swedish between 1983 and January 2016  RCTs of more than 3 months duration in adults with Type 2 diabetes  Trials must have compared a low carbohydrate diet (defined as ≤40% total energy from carbs) to a diet with more than 40% energy from carbs | 19 studies included in meta-analyses (according to their PRISMA diagram, but only 18 studies are included in the forest plots within the paper)  Two other papers (Samaha et al 2003 and Garge et al 1994) were included in this review, but were seemingly not included within any meta-analyses despite no reason for exclusion being reported | Summary information presented for the 23 RCTs included in the systematic review rather than just the 19 in the meta-analysis. 10 of these had a high risk of bias, three had a low risk, and the remaining 10 were unclear  No indication of publication bias. | When all studies were pooled, low carbohydrate diets were associated with greater overall reductions in HbA1c (−1.0 mmol/mol, 95% CI −1.9 to −0.1; I^2^ = 7%; 16 studies), though the difference was small and the result was largely driven by the results of short-term studies and by trials associated with a high risk of bias  Overall, a low carbohydrate diet was not associated with greater weight loss than a low fat diet (-0.35kg, 95%CI -0.91 to 0.21; I^2^ = 29%; 17 studies), but subgroup analysis suggested more positive results in short-term studies (3-6 months: mean difference -0.87kg, 95%CI -1.88 to 0.15; I^2^ = 33%; 7 studies) than in studies with a longer follow up (>12 months: mean difference 0.14kg, 95%CI -0.29 to 0.57kg; I^2^ = 0%; 10 studies)  Meta-analyses showed no significant difference between groups in effect on HDL-c (0.04 mmol/L, 95%CI −0.01 to 0.10; I^2^ = 72%; 16 studies), LDL-c (−0.01 mmol/L; 95%CI −0.13 to 0.11; I^2^ = 64%; 15 studies) and TC (0.04 mmol/L, 95%CI −0.12 to 0.20; I^2^ = 71%; 14 studies), but showed a slightly greater reduction in TG with a low carbohydrate diet (−0.13, 95% CI −0.24 to −0.02 mmol/L; I^2^ = 57%; 16 studies)  There was no significant difference in the effect of a low carbohydrate diet on SBP or DBP when compared to control (SBP: −0.93 mmHg, 95%CI −2.24 to 0.37; I^2^ = 0%; 14 studies. DBP: −0.21 mm Hg, 95%CI −1.20 to 0.79; I^2^ = 0%; 12 studies)  The limited information given in the included studies suggests that there was a greater reduction in the use of diabetes medication (mainly insulin) in the low carbohydrate groups, which may have masked a more positive impact on glycaemic control. However, only four studies found a significant difference in change in diabetes medication between the diets, and some of the studies repeated their analyses, adjusting for difference in medication, and found that it did not alter their conclusions  12 of the 13 studies that included relevant information reporting that there were no serious adverse events or mild adverse events such as mild hypoglycaemia. The only serious adverse advent reported in any of the included studies was not in the low carbohydrate group | High heterogeneity for many of the analyses  High threshold for classifying low carbohydrate, in relation to the definitions used within the current review. Subgroup analyses were included in the supplementary material to compare the effect of “moderate” low carbohydrate diet (30-40% total energy from carbohydrate) and “very low carbohydrate diets” (21-70g carbohydrate per day), though limited details are presented (e.g. it is unclear how many studies were included in each group – results were summarised in a table rather than presented in the form of a forest plot). There were no statistically significant differences in outcomes, in comparison to the results when all studies were included together, based on the grouping of studies in this way. |
| Huntriss et al 2018(12) | Studies published in English up to June 2016  RCTs with adults with Type 2 diabetes  The intervention group had to be a low carbohydrate diet "as stated by the author", and had to have a lower carbohydrate intake than the control group  Studies that were shorter than 48 weeks were not included in the meta-analyses | 7 studies included in meta-analysis for HbA1c, 6 studies included in meta-analysis for weight | Not presented separately for the studies included in the meta-analyses, but 15 of the 18 studies included in the review as a whole were considered high risk on at least one of the criteria | HbA1c improved significantly more in those following low carbohydrate diets (effect estimate = −0.28%, 95%CI −0.53 to −0.02, p = 0.03; I^2^ = 54%; 7 studies)  There was no difference in body weight change between diets (estimated effect = 0.28 kg, 95%CI −1.37 to 1.92, p = 0.74; I^2^ = 75%; 6 studies)  SBP improved significantly more in those following low carbohydrate diets (estimated effect = -2.74 mmHg, 95%CI -5.27 to -0.20, p = 0.03; I^2^ = 43%; 7 studies). There was no difference in DBP change between diets (estimated effect = -0.99 mmHg, 95%CI -2.24 to 0.25, p = 0.12; I^2^ = 15%; 7 studies)  HDL-c improved significantly more in those following low carbohydrate diets (estimated effect = 0.06 mmol/L, 95%CI 0.04 to 0.09, p < 0.00001; I^2^ = 1%; 7 studies)  TG improved significantly more in those following low carbohydrate diets (estimated effect = -0.24 mmol/L, 95%CI -0.35 to -0.13, p < 0.00001; I^2^ = 0%; 7 studies)  There was no difference in TC change (estimated effect = -0.08 mmol/L, 95%CI -0.23 to 0.08, p = 0.35; I^2^ = 60%; 7 studies) or LDL-c change between diets (estimated effect = 0.05 mmol/L, 95%CI -0.10 to 0.19, p = 0.54; I^2^ = 0%; 5 studies)  All 14 included studies that reported medication changes found a greater reduction in requirements in the low carbohydrate groups. Where assessed, this was statistically significant in 9/11 studies (82%) | Did not stipulate what they classed as low carb, so included studies which would not meet the definitions applied in the current review. |
| Sainsbury et al 2018(13) | Studies published in English between 1st Jan 1980 and 31st Aug 2016  RCTs comparing carbohydrate restricted diets (≤45% total energy) with high carbohydrate diets (>45% total energy) in adults with Type 1 or Type 2 diabetes | 25 studies (28 papers) included in meta-analyses (n = 2412)  [note: 14 were included as "moderate carb" diets] | Fifteen studies reported using random sequence generation, while the remaining studies did not provide sufficient information to assess this  Use of allocation concealment was poorly reported across the majority of studies (n  = 22). Due to inherent difficulties in blinding participants and personnel in dietary intervention studies, it was assumed, unless otherwise stated, that no blinding was conducted. Consequently, there was a high risk of bias across all studies for self-reported outcomes due to possible bias in patients’ self-reported dietary intake and the analysis of food records  Eight studies were classified as being at high or unclear risk for the “other biases” domain, due to stated conflicts of interest from funding sources  Overall, nine studies were classified as being at low risk, seven at high risk, and nine at unclear risk of bias  Egger’s test revealed publication bias was present at 3 months (P = 0.005) but not at 6 (P = 0.125) or 12 months (p = 0.052). | There was a significantly greater reduction in HbA1c for the low carbohydrate diet (< 26% of total energy) than the high carbohydrate diet at 3 months (WMD: -0.47%, 95% CI -0.71 to -0.23, p = 0.0001; I^2^ = 0%; 4 studies) and 6 months (WMD: -0.36%, 95% CI -0.62 to -0.09, p = 0.008; I^2^ = 0%; 5 studies)    There were no significant differences between diet groups for HbA1c change at 12 months (WMD: -0.09%, 95% CI -0.21 to 0.03, p = 0.12; I^2^ = 16%; 12 studies) or 24 months (WMD: -0.11%, 95% CI -0.38 to 0.15; 3 studies; p values and I^2^ were not reported for this time point)  At 3 months there was greater weight loss on the low carbohydrate diet (< 26% of total energy) compared to the high carbohydrate diet (WMD: - 2.47kg, 95% CI -3.33 to -1.60; number of studies, p values and I^2^ not reported for this analysis)  There was no difference between diets for weight loss at 6 (WMD: -0.14kg, 95% CI -0.94 to 0.65, p = 0.05; I^2^ = 48%; 9 studies) or 12 months (WMD: -0.43kg, 95%CI: -0.93 to 0.07, p = 0.09; I^2^ = 0%; n = 10 studies). I^2^ not reported for these analyses  Where reported, the authors stated that “there was a greater reduction in medication use for participants on carbohydrate-restricted diets compared with high carbohydrate diets at every time point. Carbohydrate restriction either reduced the dosage of oral medications and/or insulin, or saw an elimination of medication for participants across all studies that reported on medication outcomes.” Methods of measuring medication use were variable across studies | High threshold for classifying low carbohydrate, in relation to the definitions used within the current review; though analyses did sub-divide, including a "low carb" group defined as <26% total energy, mitigating for this  Due to high risk of performance and detection bias, and inconsistency in the estimates of effect across studies, the evidence of HbA1c change was graded low quality  14 of the included studies were isocaloric (between diets) by design, and thus precluded the ability to assess possible free-living benefits of included diets  Included Saslow et al 2014(10) even though not all participants had Type 2 diabetes (it is also of note that carbohydrate intake was significantly reduced in the control group of this study, from a mean of 224g/day to 160g/day) |
| Meng et al 2017(14) | RCTs up to  January 2017 in patients with Type 2 diabetes (doesn't explicitly state adults)  No publication timing or language restrictions  Low carbohydrate groups defined as less than 130g or 26% energy per day, compared to normal or high carb | 9 studies included in meta-analyses (n = 734) | 5 of 9 trials were considered high quality (based on modified Jadad score). Primary issue with the other studies was the lack of blinding and concealment of allocation  Visual inspection of funnel plots and Egger’s test suggests no evidence of publication bias for fasting plasma glucose (P = 0.28), HbA1c (P = 0.98), TC (P = 0.78), TG (P = 0.75), HDL-c (P = 0.57), LDL-c (P = 0.37), and weight loss (P = 0.80) | HbA1c decreased significantly more in low carbohydrate groups compared to control groups (WMD: -0.44%, 95% CI -0.61 to -0.26, p < 0.01; I^2^ = 20%; 9 studies)  There was no difference in the change in fasting plasma glucose between groups (WMD: -0.05 mmol/l, 95% CI -0.58 to 0.47, p = 0.84; I^2^ = 0%; 5 studies)  There was no difference in the change in body weight between groups  (WMD: -0.94 mmol/l, 95% CI -1.92 to 0.05, p = 0.06; I^2^ = 36%; 8 studies)  Low carbohydrate diets reduced TG significantly more than the control groups (WMD: -0.33 mmol/l, 95% CI -0.45 to -0.21, p < 0.01; I^2^ = 0%; 9 studies)  Low carbohydrate diets improved HDL-c significantly more than the control groups (WMD: 0.07 mmol/l, 95% CI 0.03 to 0.11, p < 0.01; I^2^ = 41%; 8 studies)  There was no difference in the change in TC (WMD: 0.06 mmol/l, 95% CI -0.08 to 0.21, p = 0.33; I^2^ = 0%; 6 studies) or LDL-c between diets (WMD: 0.04 mmol/l, 95% CI -0.08 to 0.16, p = 0.33; I^2^ = 0%; 7 studies) | Includes Iqbal et al 2010(5), where there was no difference in macronutrient intake between groups (and carbohydrate intake was actually higher in the control arm at multiple time points)  Included Saslow et al 2014(10) even though not all participants had Type 2 diabetes (it is also of note that carbohydrate intake was significantly reduced in the control group of this study, from a mean of 224g/day to 160g/day) |
| Van Zuuren et al 2018(15) | Studies published up to 21st March 2017  No explicit mention of whether there were any language restrictions  RCTs and controlled clinical trials (CCTs) which compared a low carbohydrate diet (defined as ≤40% total energy from carbs) with low fat (defined as ≤30% total energy from fat) over a period of at least 4 weeks in adults with Type 2 diabetes  Only included data from crossover trials that had incorporated wash-out periods of ≥4 wk between interventions | 17 studies included in meta-analyses (studies were divided based on their duration before meta-analyses were performed, n ranged from 42 to 539 for each analysis) | Summary information presented for all studies included in the systematic review, not just those included in meta-analyses. 19/33 RCTs were judged to be high risk and 14 had unclear risk; 1/3 CCTs had a moderate risk, 2 had a serious risk | HbA1c improved more in low carbohydrate groups in short-term (studies up to 8 weeks in duration: mean difference -1.38%, 95%CI -2.64 to -0.11, p = 0.03; I^2^ = 68%; 2 studies), medium term (8-16 weeks: -0.55%, 95%CI -0.93 to -0.17, p = 0.005; I^2^ = 54%; 4 studies; or 16-26 weeks: -0.26%, 95%CI -0.50 to -0.02, p = 0.04, I^2^ = 59%; 7 studies), and longer-term studies (≥26 weeks: -0.36%, 95%CI -0.58 to -0.14, p = 0.001; I^2^ = 0%; 4 studies). When only studies that were 2 years in duration were included however there was no difference in HbA1c change between groups (mean difference 0.02%, 95%CI -0.37 to 0.41, p = 0.93; I^2^ = 13%; 3 studies)  Fasting plasma glucose improve more in low carbohydrate groups in studies of between 3 and 12 months (-0.51 mmol/l, 95%CI -0.91 to -0.12, p = 0.01; I^2^ = 71%; 6 studies) but not in studies that were 12 months or longer (-0.37 mmol/l, 95%CI -1.22 to 0.48, p = 0.39; I^2^ = 92%; 4 studies)  Body weight reduced significantly more in the low carbohydrate group in studies lasting between 8 and 16 weeks (mean difference −2.04 kg, 95% CI −3.23 to −0.85 kg), though there was no difference at other time points. For WC and BMI there was little difference between diets at any time point  TG improved significantly more in the low carbohydrate groups in studies between 16 and 26 weeks long (mean difference -0.22 mmol/l, 95%CI -0.37 to -0.08, p = 0.002; I^2^ = 41%; 6 studies), and in long-term studies, whether this is classed as being >26 weeks (-0.25 mmol/l, 95%CI -0.47 to -0.04, p = 0. 02; I^2^ = 73%; 5 studies) or only includes studies with a duration of 2 years (-0.19 mmol/l, 95%CI -0.32 to -0.05, p = 0.007; I^2^ = 0%; 2 studies). There were no differences in studies shorter than 8 weeks, or studies of between 8 and 16 weeks in duration  HDL-c improved significantly more in the low carbohydrate groups in long-term studies, whether this is classed as being >26 weeks (mean difference = 0.11 mmol/l, 95%CI 0.05 to 0.18, p = 0.0007; I^2^ = 66%; 4 studies) or only includes studies with a duration of 2 years (0.12 mmol/l, 95%CI 0.07 to 0.17, p < 0.00001; I^2^ = 0%; 2 studies). There were no differences in studies shorter than 8 weeks, studies of between 8 and 16 weeks in duration, or studies of between 16 and 26 weeks in duration  There were no differences in changes in LDL-c between diets at any time point (12 studies)  There were no differences in changes in SBP between diets at any time point (7 studies), and for DBP the only difference was for studies lasting 6 months where there was a greater reduction for the low carbohydrate diets (mean difference−1.91 mm Hg, 95% CI −3.63, −0.18 mm Hg)  Of the 4 studies that adequately reported changes in diabetes medications, 3 demonstrated a reduced requirement in the low carb groups but not the low fat groups. Statistical analysis of this was not possible due to inconsistent methods of reporting | High threshold for classifying low carbohydrate, in relation to the definitions used within the current review  No apparent attempt to group based on target, or actual, carbohydrate intake  High heterogeneity in many of the analyses. |
| McArdle et al 2019(16) | Studies published in any language between 1976 and April 2018  RCTs including adults with Type 2 diabetes with a minimum intervention duration of 8 weeks and reported outcomes at a minimum of 12 weeks, and with the proportion or quantity of dietary carbs restricted in the intervention group  Studies also had to report a measure of actual carbohydrate intake during or at the end of the intervention | 25 studies in meta-analysis (n = 2132 for primary outcomes) [note: 12 were included as "moderate carb" diets] | The principal risk of bias stemmed either from the poor description of the randomization sequence and allocation concealment or from there being no description of the pre-study dietary intake of participants (classified under “other bias”). One or more of these biases were present in over a third of studies included in the review | Meta-analyses conducted for HbA1c for all studies found no overall effect of modifying carbohydrate (WMD: –0.09%, 95%CI –0.27 to 0.08, p = 0.30; I^2^ = 72%; 25 studies). Subgroup analysis of studies meeting the definition of very low carbohydrate (<50 g per day) also found no overall effect (WMD: –0.13%, 95%CI –0.34 to 0.08, p = 0.24; I^2^ = 19%; 8 studies, though actual mean carbohydrate intake was only below 50g in two of them). Analysis of the subgroup of low carbohydrate diet studies (50–130 g per day) showed a statistically and clinically significant result in favour of the intervention diet (WMD: –0.49%, 95%CI –0.75 to –0.23, p = 0.0002; I^2^ = 0%; 5 studies). All studies in this subgroup were of ≤6 months in duration  No overall effect on weight was observed (WMD: –0.13 kg, 95%CI –0.33 to 0.08, P = 0.22; I^2^ = 78%; 25 studies). In the low-carbohydrate subgroup there was a statistically significant pooled effect in favour of restricted carbohydrate (WMD: –0.43 kg, 95%CI –0.74 to –0.12, p = 0.006; I^2^ = 24%; 5 studies)  Of the 25 studies, 11 did not fully report outcomes for blood pressure and, in those that did, changes were “unremarkable and rarely reached statistical significance”  Blood lipid outcomes (TC, LDL-c and HDL-c) were reported in 17 of the 25 studies. Statistically significant differences between groups were seen in just seven of the studies and the most commonly observed difference was a greater increase in HDL-c in the moderate-carbohydrate group | Actual carbohydrate intake reported would not qualify multiple studies to fit in the subgroup analyses they were included in; e.g. the very low carbohydrate analyses included Davis et al 2009(17), Tay et al 2015(18) and Sato et al 2017(19) when reported intake was >50g/day  Includes Iqbal et al 2010(5), where there was no difference in macronutrient intake between groups (and carbohydrate intake was actually higher in the control arm at multiple time points). This was one of only two papers classified as very low carbohydrate that didn’t favour the intervention group  Limited reporting of outcomes for blood pressure and blood lipids.  Included Saslow et al 2017(20), for which carbohydrate intake was below the threshold for low carb at some time points  Appears to have missed papers that meet inclusion criteria*, for example: Stern et al (2004)(4); Mayer et al (2014)(21); Guldbrand et al (2012)(22) – which reports longer-term outcomes from the same study as Jonasson et al (2014) (23); and Tay et al (2018)(24) – which reports longer-term outcomes from the same study as Tay et al 2014(25) and Tay et al 2015(18). Failure to report which studies were not included and why removes the ability to assess whether these papers were omitted for a reason, compared to whether they were missed to limitations with the search strategy |
| Goldenberg et al 2021(26) | Studies published in any language up to 25^th^ August 2020  RCTs in people with Type 2 diabetes comparing low carbohydrate diets with any wait list controls or any active controls  Trials had to be 12 weeks or longer  Low carbohydrate diets were defined as less than 26% calories from carbohydrates or less than 130g carbohydrates per day | 23 studies included in meta-analyses (n = 1,357) | 18/23 studies reported missing outcome data, with 10 reporting >20% data missing. Risk of bias for missing outcome data was however classed as low for over 90%  59.4% of outcomes rated as having some concern of bias or high risk of bias (though the majority of these had “some concern of” rather than “high” risk)  “Some concerns” of bias for randomisation process in >40% trials  Low risk of bias for deviations from intended intervention in approximately 85% of trials  Low risk of bias for measurement of outcome in >90% of trials  Low risk of bias for selection of reported results in >80% of trials | When remission was defined independent of medication use, low carbohydrate diets increased remissions by 32% compared to control at 6 months (risk difference = 0.32, 95%CI 0.17 to 0.47, 8 studies, n = 264, GRADE certainty of evidence = moderate)  When remission definition required absence of medication, low carbohydrate diets increased remission by 5% compared to controls at 6 months (risk difference = 0.05, 95%CI -0.05 to 0.14, 5 studies, n = 199, GRADE = low). This was likely not significant though  When remission was defined independent of medication use, low carbohydrate diets increased remissions by 10% compared to control at 12 months (risk difference = 0.10, 95%CI -0.02 to 0.21, 3 studies, n = 171, GRADE = moderate) . This was likely not significant though  When remission definition required absence of medication, low carbohydrate diets decreased remission by 4% compared to controls at 12 months (risk difference = -0.04, 95%CI -0.16 to 0.09, 2 studies, n = 126, GRADE = low). This was likely not significant though  Patients in low carbohydrate groups achieved a greater reduction in HbA1c at 6 months (mean difference -0.47%, 95%CI -0.60 to -0.34, 17 studies, n = 747, GRADE = high)  Patients in low carbohydrate groups achieved a greater reduction in HbA1c at 12 months (mean difference -0.23%, 95%CI -0.46 to 0.00, 8 studies, n = 489, GRADE = moderate)  Patients in low carbohydrate groups achieved a greater reduction in fasting glucose at 6 months (mean difference -0.73 mmol/l, 95%CI -1.19 to -0.27, 14 studies, N = 365, GRADE = moderate), though there was statistically significant high heterogeneity for this analysis that could not be explained by analyses performed in this review  There was no difference between groups for change in fasting glucose at 12 months (mean difference 0.06 mmol/l, 95%CI -0.37 to 0.48, 6 studies. N = 611, GRADE = moderate)  Patients in low carbohydrate groups achieved greater reductions in HOMA-IR at 6 months (mean difference -0.14, 95%CI -0.51 to 0.23, 6 studies, n = 241, GRADE = very low). This was likely not significant though  Patients in low carbohydrate groups achieved greater reductions HOMA-IR at 12 months (mean difference = -0.13, 95%CI -0.39 to 0.13, 2 studies, n = 135, GRADE = very low). This was likely not significant though  Patients in low carbohydrate groups achieved greater weight loss at 6 months (mean difference -3.46kg, 95%CI -5.25 to -1.67, 18 studies, n =882, GRADE = moderate). There was high heterogeneity for this analysis, which was explained by risk of bias. In studies at low risk of bias difference was even greater (mean difference -7.41kg, 95%CI -9.75 to -5.06, 6 studies, n = 171, GRADE not reported)  There was no difference between groups for weight loss at 12 months (mean difference 0.29kg, 95%CI -1.02 to 1.60, 7 studies, n = 499, GRADE = moderate)  Patients in low carbohydrate groups achieved greater reductions in TG at 6 months (mean difference -0.30, 95%CI -0.43 to -0.17, 19 studies, n = 860, GRADE = high)  Patients in low carbohydrate groups achieved greater reductions in TG at 12 months (mean difference -0.32, 95%CI -0.51 to -0.12, 7 studies, n = 459, GRADE = high)  There was no difference between groups for TC at 6 (mean difference -0.10, 95%CI -0.41 to 0.20, 12 studies, n = 576, GRADE = moderate) or 12 (mean difference 0.11, 95%CI -0.05 to 0.27, 6 studies, n = 430, GRADE = moderate) months  There was no reported difference between groups for HDL-c at 6 (mean difference 0.06, 95%CI 0.01 to 0.10, 16 studies, n = 647, GRADE = high) or 12 (mean difference = 0.04, 95%CI 0.00 to 0.08, 7 studies, n = 458, GRADE = high) months; though as the 6 month difference’s 95%CI did not cross 0 it is likely that this difference was statistically significant in favour of low carbohydrate diets  There was no difference between groups for LDL-c at 6 months (mean difference 0.02, 95%CI -0.09 to 0.12, 15 studies, n = 672, GRADE = high)  The study reported an increase in LDL-c at 12 months (mean difference 0.14, 95%CI 0.00 to 0.28, 6 studies, n = 429, GRADE = moderate), but this was not statistically significant (P = 0.05)  There was no difference between groups for C- Reactive protein at 6 (mean difference 0.16, 95%CI -0.27 to 0.59, 7 studies, n =359, GRADE = moderate) or 12 (mean difference 0.37, 95%CI -0.44 to 1.18, 2 studies, n = 141, GRADE = very low) months  Patients in low carbohydrate groups achieved greater reductions in diabetes medication at 6 months (risk difference 0.24, 95%CI 0.12 to 0.35, 7 studies, n = 240, GRADE = moderate)  Patients in low carbohydrate groups achieved greater reductions in diabetes medication at 12 months (risk difference 0.33, 95%CI 0.00 to 0.66, 3 studies, n = 148, GRADE = low)  There was no difference between groups for the number of total or serious adverse events at 6 months (total adverse events: risk difference 0.04, 95%CI 0.1 to 0.08, 9 studies, n = 423, GRADE = low; serious adverse events: risk difference 0.00, 95%CI -0.03 to 0.02, 8 studies, n = 448, GRADE = low)  There was no difference between groups for the number of total or serious adverse events at 12 months (total adverse events: risk difference -0.05, 95%CI -0.24 to 0.14, 2 studies, n = 156, GRADE = very low; serious adverse events: risk difference -0.01, 95%CI -0.06 to 0.04, 3 studies, n = 217, GRADE = low) | Inclusion criteria allowed inclusion of studies that were highly confounded, for example by allowing studies where the intervention group participants also took part in an interval training programme(27), where the intervention was of “low-carbohydrate and protein sparing modified fast” rather than just a carbohydrate restricted diet(28) or where the control group participants were prescribed orlistat alongside dietary changes(29)  Forest plots were only reported for two outcomes in the paper (remission at 6 months and weight loss at 6 month), and no additional plots were included in the supplementary material. For one of these plots (remission) the results reported in the plot (which used risk differences) were different to the results reported in the table (which used risk ratio), the latter of which was used for informing the conclusions (as high heterogeneity was not observed for risk ratio)  Minimal details reported for analyses of secondary outcomes (including in supplementary materials), including that it did not report which studies were included for each of the analyses for secondary outcomes and heterogeneity was not reported or explored. This severely limited the ability to consider the validity and importance of these outcomes *(The secondary outcomes were HOMA-IR, all included lipid markers, c-reactive protein and medication reduction)*  Study included any data between 9 and 15 months in 12 month analyses  Includes Iqbal et al 2010(5), where there was no difference in macronutrient intake between groups (and carbohydrate intake was actually higher in the control arm at multiple time points)  Included Saslow et al 2014(10) even though not all participants had Type 2 diabetes (though this was acknowledged in the paper, and it is stated that its inclusion was consistent with decisions made *a priori;* it is however also of note that carbohydrate intake was significantly reduced in the control group of this study, from a mean of 224g/day to 160g/day)  Included Saslow et al 2017(20), for which carbohydrate intake was below the threshold for low carb at some time points  Appears to have missed papers that meet inclusion criteria*, including a number of papers that were included by McArdle et al 2020 in the most recent previous review (which had similar inclusion criteria), as well as: Stern et al (2004)(4); Mayer et al (2014)(21); and Tay et al (2018)(24) – which reports longer-term outcomes from the same study as Tay et al 2014(25) (Tay et al 2015(18) was not included either, despite also reporting longer term outcomes than the publication that was included from this study). Failure to report which studies were not included and why removes the ability to assess whether these papers were omitted for a reason, compared to whether they were missed to limitations with the search strategy |
| * throughout this table the term “low carbohydrate” is used in line with the definitions applied by the authors of the relevant systematic review, which is not necessarily aligned with the definitions used within the current review. Differences are highlighted within the “limitations” column  ** articles are listed in chronological order based on the latest date for which they included studies  WMD = weighted mean difference, 95%CI = 95% confidence intervals, HbA1c = glycated haemoglobin (reported in the units used by the authors of the relevant review), BMI = body mass index, WC = waist circumference, SBP = systolic blood pressure, DBP = diastolic blood pressure, TC = total cholesterol, TG = triglycerides, LDL-c = low-density lipoprotein cholesterol, HDL-c = high-density lipoprotein cholesterol | | | | | |

**Supplementary Table 2. Summary of randomised controlled trials comparing low carbohydrate dietary approaches (carbohydrate intake below 130g/day or 26% total energy) with any control diet in people with Type 2 diabetes (minimum 50 participants and three months duration)**

| **Article reference** | **Participants** | **Study duration** | **Intervention group diet** | **Control**  **group diet** | **Actual carbohydrate intake** | **Completion rates** | **Key outcomes** |
| --- | --- | --- | --- | --- | --- | --- | --- |
| Stern et al 2004(4) * | 54 | 12 months | <30g carbohydrate /day  No energy intake target  No protein target | Instructed to reduce caloric intake by 500 calories per day, with less than 30% of calories derived from fat  No protein target | Intervention group: 120g/day (SD 93g)  Control group: 230g/day (SD 150g) | Low carb: 18/27 (67%)  Control: 16/27 (59%) | HbA1c reduced by 0.8±1.0% (from 7.4±1.6% to 6.6±1.4%) in low carbohydrate arm, and by 0.1±1.6% (from 7.3±1.1% to 7.2±1.9%) in control arm. Between group difference was -0.7% (95%CI -1.6 to 0.2), p = 0.102  Other results (e.g. body weight and blood lipids) are not presented separately for people with Type 2 diabetes  Changes in medication were not reported  Adverse outcomes were not reported separately for people with Type 2 diabetes |
| Daly et al 2006(30) | 102 | 3 months | ≤70g carbohydrate /day  No energy intake target  No protein target | “Standard healthy eating advice”, focusing on reducing fat intake. This was combined with instructions to reduce portion size  No energy intake target  No protein target | Intervention group: 110g/day (SEM 6g)  Control group: 169g/day (SEM 10.8g) | Low carb: 40/51 (78%)  Control: 39/51 (76%) | Body weight reduced more in the low carbohydrate group (-3.55±0.63kg v -0.92±0.40kg; 95%CI of change 1.16 to 4.09kg more in low carb, p = 0.001)  TC to HDL-c ratio improved more in the low carbohydrate group (-0.48±0.11 v -0.10±0.10, 95%CI of change 0.09 to 0.68, p = 0.011)  There were no differences between groups for HbA1c, TG or SBP  In those taking exogenous insulin, dosage was reduced in approximately 85% of users in the low carbohydrate group and 22% in the control group. 16% of users in the control group increased insulin, compared to 5% in the low carbohydrate group. Use of oral hypoglycaemic agents remained unchanged in both groups  No adverse events were reported in either group |
| Westman et al 2008(31) | 84 | 6 months | <20g carbohydrate /day  No explicit energy intake target stated  No explicit protein target stated | Approximately 55% of daily caloric intake from carbohydrate  A low-glycemic index, reduced-calorie diet (500kcal less than calculated energy intake)  No explicit protein intake target stated | Intervention group: 13% total energy; 49g/day (SD 33g)  Control group: 44% total energy; 149 g/day (SD 46g) | Low carb: 21/38 (55%)  Control: 29/46 (63%) | HbA1c reduced more in the low carbohydrate group (-1.5% v -0.5%, p = 0.03)  Body weight reduced more in the low carbohydrate group (-11.1kg v -6.9kg, p = 0.008)  HDL-c improved more in the low carbohydrate group (+0.1 mmol/l v ±0 mmol/l, p < 0.05)  There were no differences between groups for fasting glucose, fasting insulin, BMI, WC, SBP, DBP, TC, TG and LDL-c.  4 individuals in the low carbohydrate group were able to omit insulin by the end of the study, compared to 1 in the control group (all 5 were taking less than 20 units at baseline). A further 4 participants in the low carbohydrate arm reduced their insulin needs, compared to 2 in the control arm. Of those taking oral hypoglycaemic medications, 95.2% in the low carbohydrate arm were able to reduce their medication; compared to 62.1% in the control group  No serious adverse events were reported in either group. No differences between groups for other side-effects (headaches, constipation, diarrhoea, insomnia or back pain) |
| Goldstein et al 2011(32) | 52 | 12 months | 25g carbohydrate /day for 6 weeks, increasing to a maximum 40g carbohydrate /day  No energy intake target  No protein target | Calorie-restricted diet (up to 1500kcal for men and 1200kcal for women)  10-20% total energy from protein | Intervention group: 85g/day (SD 35g)  Control group: 208 g/day (SD 61g) | Low carb: 14/26 (54%)  Control: 16/26  (62%) | There were no differences between groups for weight, HbA1c, fasting blood glucose, SBP, DBP , or blood lipids  At 3-months: 17/26 participants in the low carbohydrate group had reduced hypoglycaemic medications, compared to 11/26 in the control group  No adverse events were reported |
| Guldbrand et al 2012(22) ** | 61 | 6 months | 20% total energy from carbohydrate  1600 kcal for women, 1800 kcal for men  30% total energy from protein | 55-60% total energy from carbohydrate    30% total energy from fat (less than 10% from saturated fat)  10-15% total energy from protein  1600 kcal for women, 1800 kcal for men | Intervention group: 25% total energy (SD 8%) at 3-6 months  Control group: 49% total energy (SD 6%) at 3-6 months | Low carb: 26/30 (87%)  Control: 28/31 (90%) | There were no significant changes between the groups for weight, BMI, WC, HbA1c, SBP, DBP or blood lipids.  Though there was not a statistically significant difference between the groups, the improvements in HbA1c (from 58.5±10.2 mmol/mol to 53.7±10.3 mmol/mol, p = 0.004, in the low carbohydrate group and 55.6±8.0 mmol/mol to 54.7±9.7 mmol/mol, p = 0.56, in the control group) and HDL-c (from 1.13±0.33mmol/l to 1.25±0.47 mmol/l, p = 0.018, in the low carbohydrate group and 1.09±0.29 mmol/l to 1.10±0.30 mmol/l, p = 0.36, in the control group) were statistically significant in the low carbohydrate group but not in the control group  Reductions in insulin dose were statistically significantly higher in the low carbohydrate group  No adverse events were reported |
| Tay et al 2018(24) *** | 115 | 24 months | <50g carbohydrate /day  500-1000 kcal energy deficit per day  28% total energy from protein | 53% total energy from carbohydrate, with a focus on low-glycemic index foods  500-1000 kcal energy deficit per day  17% total energy from protein  <30% total energy from fat (15% monounsaturated, 9% polyunsaturated, <10% saturated) | Intervention group: 83g/day (95%CI 73 to 94g) at 22-24 months  Control group: 216g/day (95%CI 206 to 227g) at 22-24 months | Low carb: 33/58 (57%)  Control: 28/57 (49%) | Improvements in weight, body fat, fasting blood glucose, HbA1c and blood pressure were similar between groups  The difference in change in TG was statistically significant, in favour of the low carbohydrate group (mean difference 0.2 mmol/l, 95%CI -0.2 to 0.5, p = 0.001)  The difference in change in HDL-c levels was statistically significant, in favour of the low carbohydrate group (mean difference 0.1 mmol/l, 95%CI -0.2 to 0.2, p = 0.004)  The low carbohydrate group had greater reductions in diabetes medication requirements, with over twice the number of participants reducing requirements by ≥20% (22 versus 9)  No adverse events were reported |

95%CI = 95% confidence intervals, HbA1c = glycated haemoglobin (reported in the units used by the authors of the relevant study), BMI = body mass index, WC = waist circumference, SBP = systolic blood pressure, DBP = diastolic blood pressure, TC = total cholesterol, TG = triglycerides, LDL-c = low-density lipoprotein cholesterol, HDL-c = high-density lipoprotein cholesterol

* An earlier publication from the same trial was also identified: Samaha et al 2003(3)

** Although this paper includes results at 12 and 24 months the six month outcomes are reported here, as this is the latest time point at which carbohydrate intake was below the threshold to be classified as a low carbohydrate diet. Jonasson et al 2014(23) also reported outcomes from this study, but at an earlier time point

*** Two previous publications from the same trial were also identified: Tay et al 2014(25) and Tay et al 2015(18)

**References**

1. Kodama S, Saito K, Tanaka S, Maki M, Yachi Y, Sato M, et al. Influence of fat and carbohydrate proportions on the metabolic profile in patients with type 2 diabetes: a meta-analysis. Diabetes Care. 2009;32(5):959-65. doi: 10.2337/dc08-1716

2. Ajala O, English P, Pinkney J. Systematic review and meta-analysis of different dietary approaches to the management of type 2 diabetes. Am J Clin Nutr. 2013;97(3):505-16. doi: 10.3945/ajcn.112.042457

3. Samaha FF, Iqbal N, Seshadri P, Chicano KL, Daily DA, McGrory J, et al. A low-carbohydrate as compared with a low-fat diet in severe obesity. The New England journal of medicine. 2003;348(21):2074-81. doi: 10.1056/NEJMoa022637

4. Stern L, Iqbal N, Seshadri P, Chicano KL, Daily DA, McGrory J, et al. The effects of low-carbohydrate versus conventional weight loss diets in severely obese adults: one-year follow-up of a randomized trial. Ann Intern Med. 2004;140(10):778-85. doi:

5. Iqbal N, Vetter ML, Moore RH, Chittams JL, Dalton-Bakes CV, Dowd M, et al. Effects of a low-intensity intervention that prescribed a low-carbohydrate vs. a low-fat diet in obese, diabetic participants. Obesity (Silver Spring). 2010;18(9):1733-8. doi: 10.1038/oby.2009.460

6. Naude CE, Schoonees A, Senekal M, Young T, Garner P, Volmink J. Low carbohydrate versus isoenergetic balanced diets for reducing weight and cardiovascular risk: a systematic review and meta-analysis. PLoS One. 2014;9(7):e100652. doi: 10.1371/journal.pone.0100652

7. Harcombe Z, Noakes T. The universities of Stellenbosch/Cape Town low-carbohydrate diet review: Mistake or mischief? South African Medical Journal. 2016;106(12):1179-82. doi: 10.7196/SAMJ.2016.v106i12.12072

8. Fan Y, Di H, Chen G, Mao X, Liu C. Effects of low carbohydrate diets in individuals with type 2 diabetes: Systematic review and meta-analysis. Int J Clin Exp Med. 2016;9(6):11166-74. doi:

9. Snorgaard O, Poulsen GM, Andersen HK, Astrup A. Systematic review and meta-analysis of dietary carbohydrate restriction in patients with type 2 diabetes. BMJ Open Diabetes Research & Care. 2017;5(1):e000354. doi: 10.1136/bmjdrc-2016-000354

10. Saslow LR, Kim S, Daubenmier JJ, Moskowitz JT, Phinney SD, Goldman V, et al. A Randomized Pilot Trial of a Moderate Carbohydrate Diet Compared to a Very Low Carbohydrate Diet in Overweight or Obese Individuals with Type 2 Diabetes Mellitus or Prediabetes. PLoS ONE. 2014;9(4):e91027. doi: 10.1371/journal.pone.0091027

11. Korsmo-Haugen HK, Brurberg KG, Mann J, Aas AM. Carbohydrate quantity in the dietary management of type 2 diabetes - a systematic review and meta-analysis. Diabetes, obesity & metabolism. 2018;21(1):15-27. doi: 10.1111/dom.13499

12. Huntriss R, Campbell M, Bedwell C. The interpretation and effect of a low-carbohydrate diet in the management of type 2 diabetes: a systematic review and meta-analysis of randomised controlled trials. European Journal of Clinical Nutrition. 2018;72(3):311-25. doi: 10.1038/s41430-017-0019-4

13. Sainsbury E, Kizirian NV, Partridge SR, Gill T, Colagiuri S, Gibson AA. Effect of dietary carbohydrate restriction on glycemic control in adults with diabetes: A systematic review and meta-analysis. Diabetes Research and Clinical Practice. 2018;139:239-52. doi: 10.1016/j.diabres.2018.02.026

14. Meng Y, Bai H, Wang S, Li Z, Wang Q, Chen L. Efficacy of Low Carbohydrate Diet for Type 2 Diabetes Mellitus Management: A Systematic Review and Meta-Analysis of Randomized Controlled Trials. Diabetes Research and Clinical Practice. 2017;131:124-31. doi: 10.1016/j.diabres.2017.07.006

15. van Zuuren EJ, Fedorowicz Z, Kuijpers T, Pijl H. Effects of low-carbohydrate- compared with low-fat-diet interventions on metabolic control in people with type 2 diabetes: a systematic review including GRADE assessments. Am J Clin Nutr. 2018;108:1-32. doi: 10.1093/ajcn/nqy096

16. McArdle PD, Greenfield SM, Rilstone SK, Narendran P, Haque MS, Gill PS. Carbohydrate restriction for glycaemic control in Type 2 diabetes: a systematic review and meta-analysis. Diabetic Medicine. 2019;36(3):335-48. doi: doi:10.1111/dme.13862

17. Davis NJ, Tomuta N, Schechter C, Isasi CR, Segal-Isaacson CJ, Stein D, et al. Comparative study of the effects of a 1-year dietary intervention of a low-carbohydrate diet versus a low-fat diet on weight and glycemic control in type 2 diabetes. Diabetes Care. 2009;32(7):1147-52. doi: 10.2337/dc08-2108

18. Tay J, Luscombe-Marsh ND, Thompson CH, Noakes M, Buckley JD, Wittert GA, et al. Comparison of low- and high-carbohydrate diets for type 2 diabetes management: a randomized trial. Am J Clin Nutr. 2015;102:780–90. doi: 10.3945/ajcn.115.112581

19. Sato J, Kanazawa A, Makita S, Hatae C, Komiya K, Shimizu T, et al. A randomized controlled trial of 130 g/day low-carbohydrate diet in type 2 diabetes with poor glycemic control. Clin Nutr. 2017;36(4):992-1000. doi: 10.1016/j.clnu.2016.07.003

20. Saslow LR, Mason AE, Kim S, Goldman V, Ploutz-Snyder R, Bayandorian H, et al. An Online Intervention Comparing a Very Low-Carbohydrate Ketogenic Diet and Lifestyle Recommendations Versus a Plate Method Diet in Overweight Individuals With Type 2 Diabetes: A Randomized Controlled Trial. J Med Internet Res. 2017;19(2):e36. doi: 10.2196/jmir.5806

21. Mayer SB, Jeffreys AS, Olsen MK, McDuffie JR, Feinglos MN, Yancy WS. Two Diets with Different Hemoglobin A(1c) and Antiglycemic Medication Effects Despite Similar Weight Loss in Type 2 Diabetes. Diabetes, obesity & metabolism. 2014;16:90-3. doi: 10.1111/dom.12191

22. Guldbrand H, Dizdar B, Bunjaku B, Lindström T, Bachrach-Lindström M, Fredrikson M, et al. In type 2 diabetes, randomisation to advice to follow a low-carbohydrate diet transiently improves glycaemic control compared with advice to follow a low-fat diet producing a similar weight loss. Diabetologia. 2012;55(8):2118-27. doi: 10.1007/s00125-012-2567-4

23. Jonasson L, Guldbrand H, Lundberg AK, Nystrom FH. Advice to follow a low-carbohydrate diet has a favourable impact on low-grade inflammation in type 2 diabetes compared with advice to follow a low-fat diet. Annals of Medicine. 2014;46(3):182-7. doi: doi:10.3109/07853890.2014.894286

24. Tay J, Thompson CH, Luscombe-Marsh ND, Wycherley TP, Noakes M, Buckley JD, et al. Effects of an energy-restricted low-carbohydrate, high unsaturated fat/low saturated fat diet versus a high carbohydrate, low fat diet in type 2 diabetes: a 2 year randomized clinical trial. Diabetes, obesity & metabolism. 2018;20(4):858-71. doi: 10.1111/dom.13164

25. Tay J, Natalie D L-M, Thompson CH, Noakes M, Buckley JD, Wittert GA, et al. A Very Low Carbohydrate, Low Saturated Fat Diet for Type 2 Diabetes Management: A Randomized Trial. Diabetes Care. 2014;37:2909–18. doi: 10.2337/dc14-0845

26. Goldenberg JZ, Day A, Brinkworth GD, Sato J, Yamada S, Jönsson T, et al. Efficacy and safety of low and very low carbohydrate diets for type 2 diabetes remission: systematic review and meta-analysis of published and unpublished randomized trial data. BMJ. 2021;372:m4743. doi: 10.1136/bmj.m4743

27. Asle Mohammadi Zadeh M, Kargarfard M, Marandi SM, Habibi A. Diets along with interval training regimes improves inflammatory & anti-inflammatory condition in obesity with type 2 diabetes subjects. Journal of Diabetes & Metabolic Disorders. 2018;17(2):253-67. doi: 10.1007/s40200-018-0368-0

28. Vlachos D, Ganotopoulou A, Stathi C, Koutsovasilis A, Diakoumopoulou E, Doulgerakis D, et al. A low-carbohydrate protein sparing modified fast diet compared with a low glycaemic index reduced calorie diet in obese type 2 diabetic patients. Diabetologia. 2011;54:S355. doi:

29. Yancy WS, Jr, Westman EC, McDuffie JR, et al. A randomized trial of a low-carbohydrate diet vs orlistat plus a low-fat diet for weight loss. Archives of internal medicine. 2010;170(2):136-45. doi: 10.1001/archinternmed.2009.492

30. Daly ME, Paisey R, Paisey R, Millward BA, Eccles C, Williams K, et al. Short-term effects of severe dietary carbohydrate-restriction advice in Type 2 diabetes—a randomized controlled trial. Diabetic Medicine. 2006;23(1):15-20. doi: 10.1111/j.1464-5491.2005.01760.x

31. Westman EC, Yancy WS, Jr., Mavropoulos JC, Marquart M, McDuffie JR. The effect of a low-carbohydrate, ketogenic diet versus a low-glycemic index diet on glycemic control in type 2 diabetes mellitus. Nutr Metab (Lond). 2008;5:36. doi: 10.1186/1743-7075-5-36

32. Goldstein T, Kark JD, Berry EM, Adler B, Ziv E, Raz I. The effect of a low carbohydrate energy-unrestricted diet on weight loss in obese type 2 diabetes patients – A randomized controlled trial. e-SPEN, the European e-Journal of Clinical Nutrition and Metabolism. 2011;6(4):e178-e86. doi: 10.1016/j.eclnm.2011.04.003
